# Correlating the Macrostructural Variations of an Ion Gel with Its Carbon Dioxide Sorption Capacity

**DOI:** 10.3390/membranes12111087

**Published:** 2022-11-01

**Authors:** Tung Nguyen, Mona Bavarian, Siamak Nejati

**Affiliations:** Department of Chemical and Biomolecular Engineering, University of Nebraska-Lincoln, Lincoln, NE 68588, USA

**Keywords:** gas sorption, polymer, ionic liquids, carbon dioxide, synergy

## Abstract

We report on a direct correlation between the macroscale structural variations and the gas sorption capacities of an ion gel. Here, we chose 1-ethyl-3-methylimidazolium bis(trifluoromethyl sulfonyl)imide ([Emim][TF_2_N]) and poly(vinylidene fluoride)-co-hexafluoropropylene (PVDF-HFP) as the ionic liquid and host polymer, respectively. The CO_2_ sorption in the thin films of the IL-polymer was measured using the gravimetric method. The results of our experiment showed that the trend in CO_2_ uptake of these mixtures was nonlinearly correlated with the content of IL. Here, we highlight that the variations in the molecular structure of the polymers were the main reason behind the observed trend. The presented data suggested the possibility of using the composition of mixtures containing IL and polymers to realize a synergistic gain for gas sorption in these mixtures.

## 1. Introduction

Macromolecules and their mixtures are essential for the development of media used in membrane-based separation. Among various polymer mixtures, those containing ionic liquids (IL) present an interesting class of materials with many applications [1]. Over the past two decades, many studies have been devoted to investigating the properties of these mixtures [2,3,4]. Because IL are widely explored as reversible CO_2_ absorbents and their mixtures have been considered selective solvents for CO_2_ sorption [5,6], polymeric membranes containing IL gained substantial attention [7]. In the simplest form, mixing IL with polymers and casting films from these mixtures led to the development of membranes that are referred to as supported ionic liquid membranes (SILMs) [8,9].

The use of IL within polymeric domains was first explored for developing ionogels [10] and later in SILM systems [11]. Using PVDF-based SILM for CO_2_ separation, it was observed that the solubility of CO_2_ in SILM could be improved twofold when compared with that of IL [12]. When confining IL in a lyotropic liquid crystal, a similar observation was made [13]. The above observations pointed to the possibility of tuning gas sorption capacities in IL phases by mixing IL with complex fluids. Classical theory suggests that preparing such mixtures can lead to a reduction in the cohesive forces of the IL phase and in the energy required to form a cavity for the guest gas molecules [14]. Additionally, ordering the IL close to a solid wall is frequently reported and is thought to be the explanation for the enhanced solubility of gas in IL in confinement [15,16,17,18,19,20].

Herein, we report on the significance of the composition of a well-known polymer gel composed of poly (vinylidene fluoride-co-hexafluoropropylene) and 1-ethyl-3-methylimidazolium bis(trifluoromethyl sulfonyl)imide on its CO_2_ sorption capacities. We note that the swelling of polymers and configuration changes that led to the structural variation in polymers were responsible for the observation of a 1.5-fold enhancement in the CO_2_ sorption in these mixtures. By controlling the composition of the mixture, we demonstrated a clear macrostructure-to-function relationship for CO_2_ solubility in these mixtures. Utilizing carbon-based additives as nucleating agents, we further explored the role of crystallinity and structure of the samples in CO_2_ sorption. The results suggested that the suppression of the β-phase in the polymer had a pronounced effect on the CO_2_ sorption capacity of the samples.

## 2. Materials and Experiments

### 2.1. Materials

The imidazolium-based ionic liquids (1-ethyl-3-methylimidazolium bis(trifluoromethyl sulfonyl)imide ([Emim][TF_2_N]) with a purity of ≥98% and poly(vinylidene fluoride-co-hexafluoropropylene) (PVDF-HFP)) with an MW of ~400,000 g/mol and triethyl phosphate (TEP) with a purity of ≥99% were purchased from Sigma-Aldrich (St. Louis, MO, USA). Graphene nanoplatelets (GNPs; Grade 4, purity > 99%, average platelet size > 2 µm, average thickness ~8–15 nm, surface area ~500–700 m^2^/g, CAS Number #7782-42-5) were purchased from Cheap Tubes Inc (Grafton, VT, USA). A carbon dioxide gas tank (CO_2_, Coleman Instrument grade, purity > 99.99%) was acquired from Matheson Tri-Gas, Inc (Lincoln, NE, USA). The polished 6 MHz Gold AT quartz crystals (QCs) were purchased from Phillips Tech (Greenville, NC, USA). The quartz crystal microbalance (QCM) sensor (Phoenix Temperature Monitoring Sensor System with an Eon-LT Monitor) was purchased from Colnatec Inc. (Gilbert, AZ, USA). The silicon wafer (type P, boron dopant (B), resistivity ~0.005–0.02, thickness ~500 µm, virgin test grade, orientation of <100>, diameter of 5.08 cm, single-side polished) was purchased from the University Wafer (South Boston, MA, USA).

### 2.2. Sample Preparation

Mixtures of polymers and ionic liquids with different compositions were prepared for the study of CO_2_ sorption. First, a mixture of 10 wt % PVDF-HFP in TEP was prepared. The mixture was heated to 100 °C and stirred at 400 rpm for 24 h. Then, the solution was cooled to room temperature. The polymeric mixtures of PVDF-HFP with 10, 30, 40, 45, 50, and 55 wt % of [Emim][TF_2_N] were prepared by adding a stock solution to the 6-dram capped glass vials (VWR, Radnor, PA, USA, Borosilicate Glass Vials). Then, the mixture was weighed on an analytical balance (Sartorius, Bohemia, NY, USA, MSA225P100DI Cubis Analytical Balance) and a known amount of IL was added to each solution to obtain the desired composition. All mixtures were stirred and heated at 310 rpm and 95 °C on a hotplate (Heidolph Inc., Wood Dale, IL USA) for 24 h before they were further used in an experiment. The three main mixtures used for the sorption study contained 10, 30, and 50 wt % of IL in the mixture, from which the mixture of 50 wt % was chosen to investigate the effect of graphene nanoplatelets (GNPs). The mixtures containing 10, 30, 40, 45, 50, and 55 wt % of IL were used to establish a calibration line for the composition. For a mixture of PVDF-HFP/ionic liquids/graphene nanoplatelets, the polymeric mixture of PVDF-HFP/GNP was first prepared. Briefly, different amounts of GNPs were weighed and transferred to the vials, then polymer solutions with known compositions were added. The mixtures were stirred and heated for 24 h at 310 rpm and 95 °C, respectively. In the second step, to reach a target composition of 50 wt % of IL, a known amount of IL was added to the vial. The mixtures were additionally stirred and heated for 24 h at 310 rpm and 95 °C, respectively.

For the thin-film preparation, the solutions were heated and stirred at 95 °C and 310 rpm, respectively, for 24 h. Spin casting of solutions was performed on various substrates such as silicon wafers, QCM substrates, and microscope glass slides; the details of the spin-coating conditions are given in Section S1.3 of the Supplementary Materials. Specifically, silicon wafer substrates were used for the polarized microscope and growth rate measurements, QCM substrates were used for the CO_2_ capture, and glass slide substrates were used for the Fourier-transform infrared spectroscopy (FTIR) measurement. The substrate was first heated at 60 °C for half an hour and then left for 71.5 h at 110 °C.

Similar to the thin-film preparation, to prepare thick films, all solutions were heated and mixed at 95 °C and 310 rpm, respectively, for 24 h. Solutions were then poured directly into aluminum-based casting wells with a 1 cm diameter and a 1.5 mm depth. The following steps were taken to cast the films: the cast polymer was transferred to a heating vacuum oven preset at 60 °C; then, the oven temperature was ramped to 90 °C at a ramp rate of 1.3 °C/min and the wells were left in the oven for two hours and 30 min. Then, the oven temperature was increased to 150 °C at a ramp rate of 1.4 °C/min and the samples were heated for 24 h to remove the residual TEP. At this point, the oven temperature was decreased to 110 °C. It was evacuated using a rotary vane pump and then the drying process continued for another 72 h. The samples were kept in the vacuum oven until they were used for measurement. The thick films were prepared for the X-ray diffraction (XRD) measurement.

### 2.3. Characterizations

The macroscale structural variations in the films were evaluated by observing the samples under an Olympus BX51 polarizing microscope equipped with a Mettler Toledo FP900 thermal system with a temperature range of room temperature to 375 °C. Additionally, the films’ topography was evaluated using atomic force microscopy (AFM). The AFM was performed using a Bruker Dimension Icon AFM (Billerica, MA, USA) in tapping mode with a range of 15 µm. The Bruker Dimension Icon AFM was operated under ambient conditions using a commercial silicon microcantilever tip on a nitride lever in ScanAsyst-Air mode. Both height and in-phase images were obtained using a scan rate of 0.988 Hz and 512 samples/line. The degree of crystallinity of the films was evaluated by using the X-ray diffraction (XRD) method. The XRD measurements were performed on Rigaku SmartLab (Rigaku Co., Tokyo, Japan).

The measurement of CO_2_ absorption was conducted by using both dynamic and static methods. The dynamic method was adopted from a previous report [21]. A high-pressure chamber equipped with a quartz crystal microbalance (QCM) was used to measure the gas sorption within the film using the gravimetric. The static method was a variation of the pressure drop approach [22,23]. Because obtaining reliable data for IL via the dynamic approach was challenging, we only relied on the results from the static method and compared those results against the reported data in the literature [24].

Before each experiment, the QCM’s placeholder was cleaned with acetone and purged with nitrogen gas to remove solvent residues. The test samples that were spin-coated on the QCM were weighed on an analytical balance (Sartorius, Bohemia, NY, USA, MSA225P100DI Cubis Analytical Balance), and its weight was recorded with two digits past decimal point. Then the samples were loaded into the QCM placeholder. Initially, the system was evacuated using a rotary vane pump and the chiller’s temperature was set. When the frequency of the coated film on the QCM was stabilized (~3.5 h in continuous vacuum), the frequency and temperature of the module were logged via Eon-LT software. The pressure of chamber was also recorded in LabVIEW. Then, the pressure was set on the pressure regulator and CO_2_ was introduced into the system. The ranges for pressure and temperature set points were between 50 and 250 psi and 10 and 40 °C, respectively. For a desorption, the QCM sensor was heated at 60 °C for 20 min while the system was continuously evacuated.

## 3. Results and Discussion

**Figure 1:**
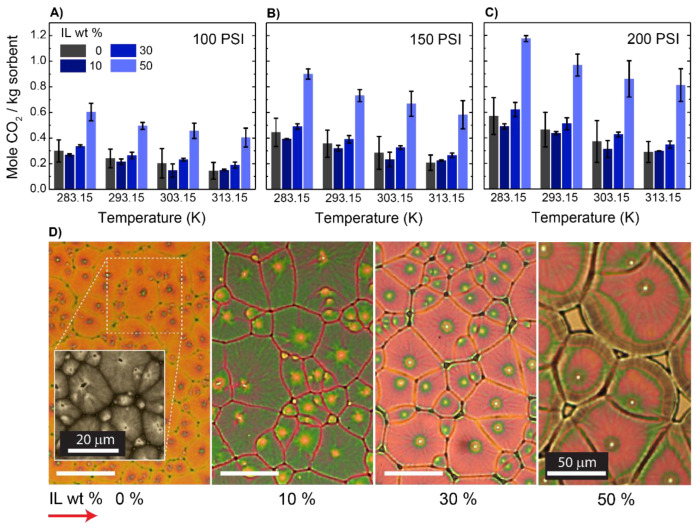
The effect of adding IL on the equilibrium absorption capacity of PVDF-HFP:IL mixtures at three different pressures of (**A**) 100, (**B**) 150, and (**C**) 200 psi. Each data point is the average of four measurements presented with one standard deviation. (**D**) Polarized microscopy images showing the spherulites grown from different mixtures with various IL content. A scale bar is applicable to all subfigures.

The CO_2_ absorption capacities of the polymer–IL films were measured; the data are presented in Figure 1A–C. The average values for the CO_2_ uptake slightly decreased as we increased the IL content of the mixture; however, this trend was reversed when the IL content was above 30%. In this range, the increase in CO_2_ uptake was nonlinearly correlated with the IL content. For example, for a mixture of 50 wt % IL in the polymer at 200 psi and 10 °C, the specific molar sorption was 1.18 ± 0.06 mol/kg while the molar sorption for the polymers and IL were 0.6 ± 0.08 and 1.28 ± 0.08 mol/kg, respectively. When further examining the data, we noted that the measured values for CO_2_ sorption in the polymer samples had a large uncertainty when compared with the CO_2_ sorption values for the samples containing IL. Given that the PVDF-HFP was a semicrystalline polymer, we attributed the standard deviation in the CO_2_ sorption in the polymer to the polymorphism of the samples [25]. In the samples that contained [Emim][TF_2_N], which is a known plasticizer of PVDF-HFP, this variability was not pronounced. This minor variability was attributed to the role of IL in suppressing film crystallinity. It was also reported that the addition of salts to PVDF-HFP favored the formation of one polymorph over the others, which loosely translated into having a more homogenous film compared to the neat polymer.

To gain further insight into the effects of the composition of a mixture on the film structure, we conducted optical microscopy. PVDF-HFP is well known for demonstrating birefringence under polarized light [26]; therefore, we used a polarized microscope for this purpose. Figure 1D presents the morphology of polymeric films as a function of their compositions. As shown, a birefringence of PVDF-HFP appeared as the weakest compared to other samples. As the content of IL in the film was increased, a stronger birefringence was apparent and the spherulitic domains became more discernible. This change matched the data reported in the literature and was attributed to the preferential assembly of the polymer lamellae into the edge-on configuration [27,28]. Additionally, in Figure 1D shows that with an increase in the concentration of the IL in the film, the spherulites became larger in size. Here, IL molecules swelled polymers and altered the polymer structures. Thus, larger spherulites were observed.

**Figure 2:**
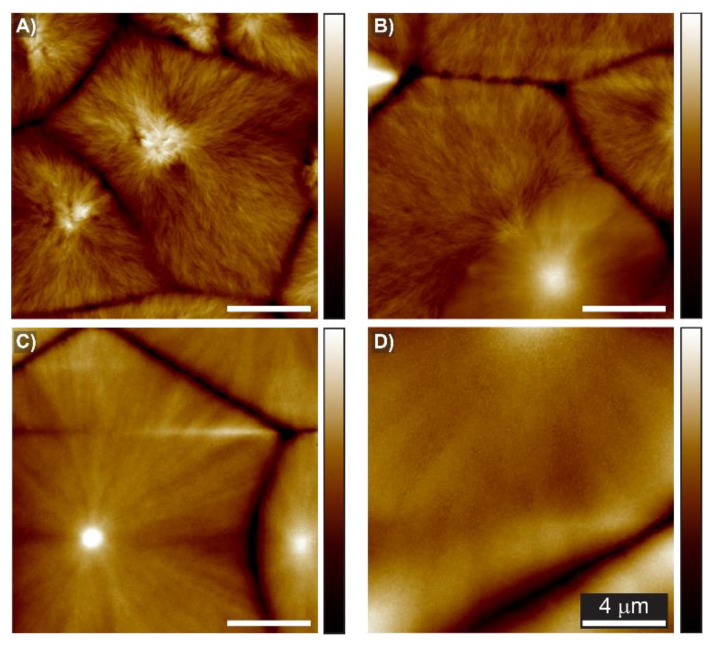
AFM height images of the sample spin-casted from different solutions of polymer–IL: (**A**) 0 wt % IL; (**B**) 10 wt % IL; (**C**) 30 wt % IL; (**D**) 50 wt % IL. A scale bar is applicable to all figures.

To further probe the film structure at the submicron level, we conducted atomic force microscopy (AFM) experiments; these data are presented in Figure 2 and the details of the sample preparation are described in Section S3.1.2 of the Supplementary Materials. As shown in Figure 2A, the morphology of the PVDF-HFP consisted of dendrites composed of small short multibranched structures, which are typically associated with flat-on lamellae [29]. The lamellae originated from spherulite centers and aggregated into small fibrils in dense clusters. When the IL content was increased to 10 wt % (Figure 2B), the dendrite-like patterns were replaced by refined and tiny fibrils. The fibrils grew parallel to each other and formed a stack of fibrils near the nuclei center. To better visualize this variation, the in-phase images are presented in Figure S7 of the Supplementary Materials. By increasing the IL content above 30%, the tiny fibrils from the spherulites’ center became omnipresent (see Figure S7). These results suggested that the addition of IL favored the formation of edge-on oriented lamellae [28,30], which led to a transition in the polymer’s structure that explained the changes in the birefringence and swelling, as shown in the optical images presented in Figure 1D.

**Figure 3:**
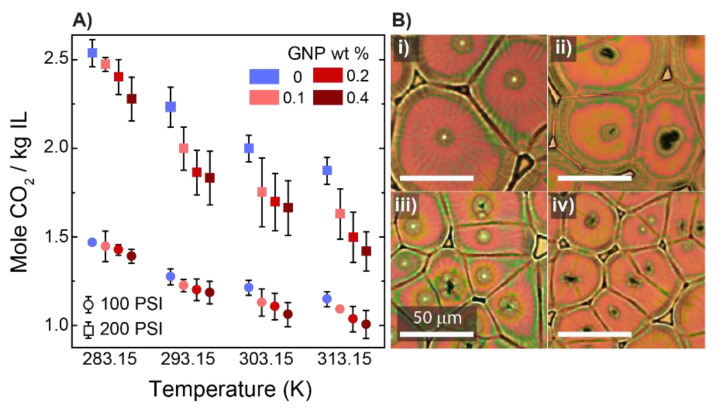
The effect of graphene nanoplate concentration on the properties of thin films. (**A**) The equilibrium absorption capacity of films at two different pressures of (⧮) 200 and (⧲) 100 psi. (**B**) Structural variation observed under polarized microscopy. Here, the concentrations of GNPs were: (i) 0, (ii) 0.1, (iii) 0.2, and (iv) 0.4 wt %. The control sample was a mixture of 50 wt % PVDF-HFP:IL to which various amounts of GNPs, as noted, were added. The error bar in Figure A represents the average values of CO_2_ uptake for five different samples presented with one standard deviation. A scale is applicable to all panels of Figure 3B.

Motivated by the previous works on the effect of the inclusion of carbon allotropes within IL and polymer phases [31,32], we used graphene nanoplatelets (GNPs) as an additive to our mixtures and probed the variations in CO_2_ uptake and the film structures. For the control experiment, we chose 50 wt % IL in the polymer and to this mixture added a small amount of GNPs ranging between 0.1 and 0.4 wt %. Figure 3A shows the CO_2_ absorption capacity for the different mixtures. For pressures of 100 and 200 psi, we observed a monotonic decline in the absorption capacity of the mixtures as a function of their GNP content, suggesting that CO_2_ interaction with the film was weakened as the GNP content was increased. As with the polymer–IL mixtures, we evaluated the morphology of the films under a polarized microscope to elucidate the effect of GNP content. Figure 3B(i–iv) present polarized images of samples prepared with different amounts of GNPs ranging from 0 to 0.4 wt %. The figure shows that when GNPs were added to the polymeric mixtures, the density and size of the spherulitic domains is changed. This observation matched the one reported in the literature [33].

**Figure 4:**
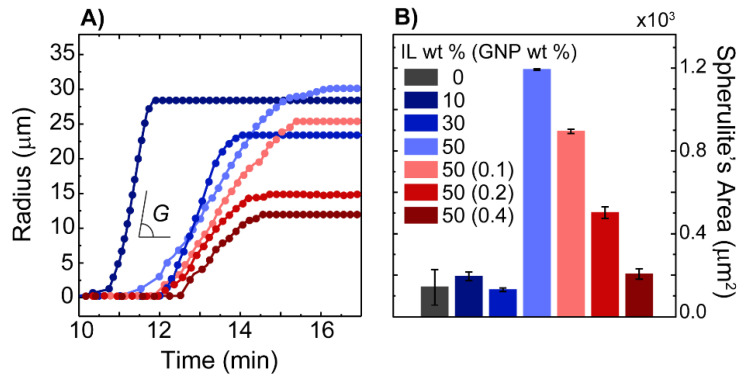
(**A**) Plot of the radii of spherulites vs. time used to estimate the spherulite growth rate (G = tan^−1^ ∡). (**B**) Average values for the area of spherulites; each bar is the average of three measurements presented with one standard deviation.

To establish a relationship between the structures and CO_2_ absorption capacities of the polymeric films, we studied the thin films’ structures and properties using different characterization methods. We first examined the growth rate and average size of the spherulites grown from different mixtures. The spherulite growth rate was obtained from the slope of the plots of radii of spherulites as a function of time, which is illustrated in Figure 4A. The size of the spherulitic domains, as shown in Figure 4B, was determined using the polarized optical images shown in Figure 1D and Figure 3B–E; more details on the experimental preparations and calculations are available in Section S3.2 of the Supplementary Materials. As shown in Figure 4A, we observed that the content of IL and GNP in the mixtures governed the growth rate and size of the spherulites. When the concentration of IL was increased to 30 wt %, the nucleation of the spherulites was delayed; increasing the IL content further to 50 wt % shifted the onset time of the growth backward. We attributed this change to the plasticization effect of IL; the addition of IL beyond the swelling capacity of polymer [34,35] enhanced the mobility of the PVDF-HFP chain while the latter led to an enhanced nucleation rate for the spherulites [36]. In contrast, the growth rates were depreciated.

Concurrent with these changes, as shown in Figure 4A, we noted that the slower growth kinetics of the polymer in mixtures containing only the polymer and an IL led to the formation of larger spherulites. Figure 4B and Figure 1D clearly demonstrate these changes. A reduction in the growth rate is often associated with the thermal mobility of chains, resulting in a longer time for the chains to fold into lamellae. This phenomenon facilitates branching and reduces the macroscopic growth rates [37]. Our calorimetry data, which are presented in Section S3.5 of Supplementary Materials, supported this point. In contrast to the polymer–IL mixtures, for the GNP-containing mixtures, a reduction in the spherulite growth rate as a function of the GNP content was attributed to the existence of GNPs and their agglomerates within the polymers, which restrained the mobility of the polymer chains. This trend has been reported for other nanocomposites [38]. When GNPs were introduced to the mixtures, the required activation energy for the chains to pack from the surface significantly increased [39]. The interaction between the partial positive charge on the C-H bonds of the PVDF-HFP and the negative charge on the surface of the GNPs created a higher free-energy barrier for nucleation, which slowed the crystallization kinetics of the polymer chains [40]. However, when the GNP content was increased, the increase in the nucleation density led to the indiscriminate growth of lamellae. As a result, we observed a larger number of spherulites with smaller sizes. This behavior was in line with a previous observation [41].

**Figure 5:**
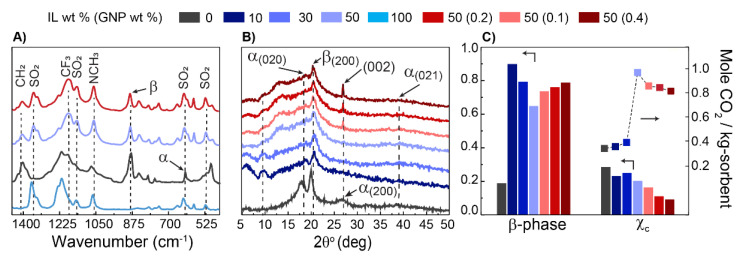
(**A**) Fourier-transform infrared spectroscopy (FTIR) fingerprint of polymer, IL, and two different mixtures cast on QCM substrate. (**B**) X-ray diffractograms of films cast from different mixtures. (**C**) The degree of β-phase and crystallinity of the samples and the normalized CO_2_ absorption capacity of thin films for a sample tested at 200 psi and 313.15 K.

To gain more information on the films’ compositions, we analyzed the films using Fourier-transform infrared spectroscopy (FTIR) and X-ray diffraction (XRD). Figure 5A presents a comparison of the IR spectra of the polymeric films. The assignment of the vibrational bands is presented in Section S3.3 of the Supplementary Materials. The characteristic band at the wavenumbers of 612 and 875 cm^−1^ were assigned to the α-phase and β-phase of the PVDF-HFP, respectively. Additionally, three distinct bands were assigned to the SO_2_ vibration of the IL. The peak appeared at 1348 cm^−1^, which corresponded to an antisymmetric SO_2_ vibration mode of the IL; this was used to estimate the IL content of the films. The procedure to estimate the composition of polymer–IL film using the FTIR signal is reported in Section S3.3 of the Supplementary Materials. Figure 5B presents the XRD pattern of these films; the predominant peaks at 2θ were equal to ~18°, 20°, and 27°, which corresponded to the α-phase (020), β-phase (200), and α-phase (200) of the PVDF-HFP, respectively [41,42]. The small broad peak at 38° corresponded to the α-phase (021) diffraction [43]. The peak at 27.5° represented π–π spacing of the GNPs [44]; this diffraction peak became stronger as the concentration of GNP was increased. A clear change in the diffractograms was observed as the compositions were varied. We attributed this change in the structure of the polymer to the strong van der Waals (vdW) interaction between the imidazolium cations of the IL and the negative dipoles of the CF_2_ groups of the PVDF-HFP, which controlled the crystallization kinetics and stabilized the formation of the α-phase of the PVDF-HFP [45,46].

Additionally, we estimated the degree of crystallinity and the β-phase of the films; the detailed procedures are presented in Section S3.4 of the Supplementary Materials. As shown in Figure 5C, increasing the IL content led to a reduction in the β-phase, which was in line with a previous report [47]. Furthermore, increasing the IL content enhanced the flexibility of the polymer chains and resulted in a reduction in the degree of crystallinity of the PVDF-HFP [42]. The latter was confirmed by the broadening of the XRD peaks, which was an indication of an increase in the volume of the disordered domains within the films [48,49]. Notably, the addition of GNPs led to a reduction in the degree of crystallinity but favored the formation of the β-phase. Here, we expected that the interaction between partial positive charges on the C-H bonds of the PVDF-HFP and the negatively charged surfaces of the GNPs led to a higher probability of the formation of “all-trans” segments of the PVDF-HFP [50,51]. As shown in Figure 5C, a reduction in the crystallinity of the polymer–IL films initially led to an increase in the CO_2_ absorption capacity of the films. Upon the addition of GNPs, although the degree of crystallinity was further reduced, the trend of the CO_2_ absorption was reversed. In this case, it was expected that the GNPs would induce a variation in the dispersion of the IL within the polymer matrix, weakening the solvation interactions. A similar observation when dealing with GNPs in complex fluid mixtures suggested that the addition of GNPs weakened the interactions between the components of the mixtures [39]. Here, we believe that the observed configurational changes of the PVDF-HFP due to the addition of GNPs resulted in an increase in the effective cohesion of the IL phase through reducing the polymer–IL interaction. As a result, the CO_2_ absorption in these films was decreased.

**Figure 6:**
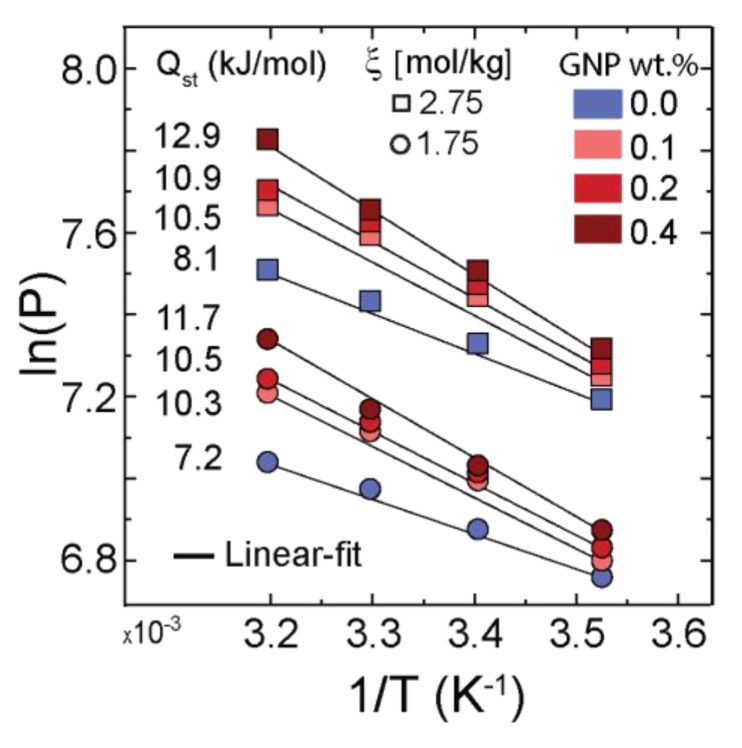
A linear plot of ln(P) versus 1/T at two excess molar concentrations (ξ) of (○) 1.75 and (□) 2.75 mol CO_2_ per kg IL; the estimated isosteric heat of absorption in kJ/mol for each composition is reported.

To gain more quantitative information about the CO_2_ sorption in the films, we estimated the enthalpies of absorption of the CO_2_ in the polymeric films. Figure 6 presents the variations in the natural logarithm of pressure at steady states as a function of the inverse temperature for a 1.75 and 2.75 equivalent excess molar concentration of CO_2_ (mole CO_2_ per kg of IL). The isosteric enthalpies of absorption were estimated from the linear fits to the Clausius–Clapeyron equation [52] and are presented in the graph; additional data at different mole uptake are presented in Section S2.3.3 of the Supplementary Materials. Here, at the constant excess molar concentration of CO_2_, a systematic increase in the enthalpy of absorption as a function of the GNP concentration was noted. From a design perspective, sorbents that require a higher heat of regeneration (enthalpy of absorption) and provide a lower capacity for gas absorption are not attractive [53]; however, the observed trends pointed to the sensitivity of the configurational properties of these mixtures to their compositions.

## 4. Conclusions

In summary, we demonstrated the effects of the macrostructures of an ion gel on its CO_2_ sorption capacity as a function of its composition. We observed a significant enhancement in the CO_2_ sorption capacity for the IL in the polymer phase. The CO_2_ sorption increased nonlinearly with increasing IL content. This nonlinearity of the CO_2_ sorption was not only based on the hole-filling process, but it was also strongly influenced by both interactions of CO_2_–Polymer–IL and the swelling behavior of the polymer. In addition, we observed a strong impact of an addition of a carbon allotrope; even at a small mass fraction, this addition led to a structural change at the macroscale that reduced the CO_2_ sorption capacity. Finally, the latter result of the heat of sorption highlighted the nonideality of these mixtures and the opportunity to choose the mixture composition as a design parameter with macroscopic fingerprints and tune the gas sorption properties of these mixtures.

## Data Availability

The data presented in this study are available on request from the corresponding author.

## Supplementary Materials

The following are available online at https://www.mdpi.com/article/10.3390/membranes12111087/s1, Figure S1: (A) FTIR fingerprint of polymeric films; the red box indicates the range used for developing a calibration curve for estimating films’ composition; (B) the established calibration curve for estimating the film composition cast from known mixtures; Figure S2: A schematic of an experimental apparatus for the dynamic method. The QCM flange shown in this figure only displays for the representative. Please refer to an experimental apparatus for an accurate dimension. Figure S3: Typical pressure and frequency traces, collected from the QCM module. Here the data is collected at 10 °C and the pressure set points were 100,150, 200 psi. (A) Bare and (B) coated QC with 50:50 wt % PVDF-HFP:IL; Figure S4: (A) A schematic of the gas manifold and the component of the apparatus used for measurement using static method. (B) Typical pressure trajectory gathered from the cell. The pressure approached a plateau after ~10 h; Figure S5: The effect of adding IL on the equilibrium absorption capacity of PVDF-HFP:IL (GNP) mixtures at three different pressures of (A) 100, (B) 150, and (C) 200 psi. Each data point is the average of four measurements presented with a standard deviation. (D) Polarized Microscopy Images showing the spherulites grown from different Polymer/IL mixtures with various GNP content; Figure S6: Enthalpy of adsorption of polymeric mixtures. (A) PVDF-HFP:IL with various concentrations of IL. (B) Polymeric mixtures of 50:50 PVDF-HFP:IL with various concentrations of GNP; Figure S7: AFM inphase images of the sample spin-cast from different solutions of Polymer:IL as specified in the figure: (A) 0 wt. % IL, (B) 10 wt. % IL, (C) 30 wt. % IL, (D) 50 wt. % IL. Arrows indicates the lamella direction: a yellow arrow indicates the orientation of fibrils of the edge-on lamellae, while a red arrow indicate the flat-on filbrils; a white arrow indicates the small fibrils; Figure S8: An overview PLM analysis is performed in 4 steps: (1) pre-processing image by scaling and selecting an image region; (2) an image classifier model is applied to obtain a resulted image from the “Trainable Weka Segmentation”; (3) an image threshold process is applied to a selected class based on users’ defined classes; (4) binary convert process is applied to separate segmented regions whereas white color corresponds to a selected region. (A) an original PLM image. (B) a scaled image. (C) an image after the training was completed. (D) trained images with separated labels were created. (E) a binary image for post-processing; Figure S9: (A) Evolution of spherulites from a thin film of 50:50 PVDF-HFP:IL coated on a silicon wafer and heated with a heating rate of 2 °C/min; the red line indicates the radius of the selected spherulite. All figures have the same scale. (B) Spherulite growth rate curve. Each data point; Figure S10: (A) FTIR spectra of polymeric mixtures; the red box indicates the frequency region, containing α-, β-, and ɣ-phase, which is used to determine the formation of ɣ-phase for the quantification of the degree of β-phase, the purple box indicates the frequency region used for the quantification of the degree of β-phase. (B) region in 1100–1450 cm^−1^, containing α-, β-, ɣ-phase; Figure S11: XRD Analysis of polymeric mixtures with various concentrations of IL and GNP. IL wt % in PVDF-HFP—(A) 0 wt %. (B) 10 wt %. (C) 30 wt %. (D) 50 wt %; GNP wt % in 50:50 PVDF-HFP:IL—(E) 0.1 wt %. (F) 0.2 wt %. (G) 0.4 wt %; Figure S12: (A) DSC curve analysis of PVDF-HFP. (B) DSC curve of Polymer:IL (GNP); Figure S13: (A) Heat Flow vs. Temperature (°C) curve analysis of PVDF-HFP. (B) Heat Flow vs. Temperature (°C) curve of Polymer:IL (GNP) during a cooling process; Table S1: Conditions for the spin-coating method; Table S2: The static gravimetric isotherm sorption results of [EMIM][TF_2_N] (Mole CO_2_ per kg IL) at 4 different pressures (psi) and temperatures (K). At 100 and 150 psi, an average and standard deviation were reported based on 3 different prepared tests. At 200 psi, an average and standard deviation were reported based on 2 different prepared tests. Data is compared with interpolated reported values; Table S3: Sorption isotherms dataset of 50:50 Polymer:IL at different temperatures and 11 different pressures. The data are the average of five different measurements presented with one standard deviation; Table S4: The enthalpy of adsorption of 50:50 Polymer:IL at different temperatures and 1.75 mole CO_2_ / kg IL. The data are the average of five different measurements presented with one standard deviation; Table S5: Spherulite growth rate of polymeric mixture with various IL and GNP contents. The average and standard deviation of all samples were reported from two spherulites that appeared first on the surface; Table S6: Characteristic FTIR peaks of polymeric film; Table S7: Defined peak locations for the peak convolution; Table S8: Relative fraction of β-phase of polymeric films. An asterisk (*) indicates that a value is estimated from an interpolation; Table S9: Parameters applied to AMORPH simulation to calculate the crystallinity and amorphousity of the polymeric film; Table S10: Pattern XRD angles of the polymeric film; Table S11: The degree of crystallinity of polymeric films. For the films with 0, 10, and 50 wt % IL content. The “Half-width (modelling)” indicates the standard deviation of how close the model to an actual dataset is; Table S12: Melting temperature of various films. For the films with 50 wt % IL content the data are representative of average values of three measurements from different samples, presented with one standard deviation; Table S13: Melting temperatures, crystallization temperatures, and solidification of various films. For the films with 50 wt % IL content, the data are representative of average values of three; Table S14: Degree of crystallinity of Polymer:IL (GNP). References [54,55,56,57,58,59,60,61,62,63,64,65,66,67,68,69,70,71,72,73,74,75,76,77,78,79,80,81,82,83] are cited in Supplementary Materials.
