# Supplementary material for: Correlating the Macrostructural Variations of an Ion Gel with Its Carbon Dioxide Sorption Capacity
Source: Membranes (Basel). 2022 Nov 1;12(11):1087. doi: 10.3390/membranes12111087 (PMC9694987; doi:10.3390/membranes12111087)
Supplement: Supplementary file 1 [file membranes-12-01087-s001.zip › membranes-1966665-supplementary.pdf]

membranes

Supplementary Materials

Correlating the Macrostructural Variations of an Ion Gel with Its Carbon Dioxide Sorption Capacity

Tung Nguyen, Mona Bavarian and Siamak Nejati *

Department of Chemical and Biomolecular Engineering, University of Nebraska-Lincoln, Lincoln, NE 68588, USA
* Correspondence: snejati2@unl.edu

Citation: Nguyen, T.; Bavarian, M.; Nejati, S. Correlating the Macrostructural Variations of an Ion gel with its Carbon Dioxide Sorption Capacity. *Membranes* **2022**, *12*, 1087. https://doi.org/10.3390/membranes12111087

Academic Editor: Isabel C. Escobar

Received: 27 September 2022
Accepted: 23 October 2022
Published: 1 November 2022

Publisher's Note: MDPI stays neutral with regard to jurisdictional claims in published maps and institutional affiliations.

Copyright: © 2022 by the authors. Licensee MDPI, Basel, Switzerland. This article is an open access article distributed under the terms and conditions of the Creative Commons Attribution (CC BY) license (https://creativecommons.org/licenses/by/4.0/).

S1. Experimental Methods

S1.1. Thick Film Preparation

Before casting, for 24 h, all solutions were heated and mixed at 95 °C and 310 rpm, respectively. To prepare a thick film, a solution was directly poured into aluminum-based casting wells, with 1 cm diameter and 1.5 mm depth. The following steps were taken to cast films; the cast polymer was transferred to a heating vacuum oven, preset at 60 °C; the oven temperature was ramped to 90 °C with a ramp rate of 1.3 °C/min, and the wells were left in the oven for two hours and 30 min. Then, the oven temperature was increased to 150 °C with a ramp rate of 1.4 °C/min and the samples were heated for 24 h to remove the residual TEP. At this point, the oven temperature was decreased to 110 °C, it was evacuated using a rotary vane pump, and the drying process continued for another 72 h. The samples were kept in the vacuum oven until they were used for measurement.

S1.2. Film Composition

To establish a correlation between the composition of the spin-coated films and the mixtures used for casting, six different compositions of Polymer:IL of 10, 30, 40, 45, 50, and 55 wt % were prepared and used for spin-coating. Figure S1a shows the normalized FTIR signal of the films cast on the glass slide. Here, we used the peak area located in between 1307 to 1370 cm^{-1} and utilized the strong sulfonyl vibration of IL at 1330 cm^{-1} to deconvolute the signals. Figure S1b shows the deconvoluted peaks used to establish the calibration line presented in Figure S1b. Each data point is representative of three experiments using the same casting solution.

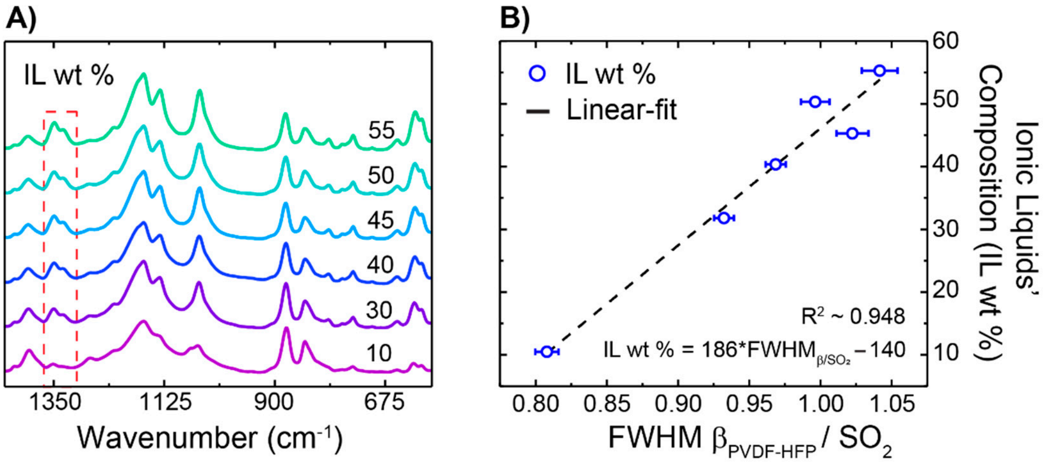

Figure S1. (**A**) FTIR fingerprint of polymeric films; the red box indicates the range used for developing a calibration curve for estimating films' composition; (**B**) the established calibration curve for estimating the film composition cast from known mixtures.

The peak deconvolution was performed, using the OriginPro software (version 9.4). Briefly, the peak fitting was done by correcting the baseline using Shirley's baseline; two peaks at 1331 and 1348 cm^{-1}, corresponding to the β-phase peak (PVDF-HFP) and SO_2 peak (IL), respectively,[1,2] are chosen for the deconvolution purpose. For the "Fit Control" parameters, the Gaussian method is applied, with the fixed base, while other parameters are adjusted to fit the data. Here we used 200 iterations and tolerance of 1×10^{-6} for variations for the residual error.

S1.3. Spin-coating parameters condition

To spin coat a thin film, the substrates were placed on the chuck of a spin coater (, Laurell Technologies Corporation, North Wales, PA, USA, Laurell H6-8) and the conditions were set accordingly. The setting parameters for the spin coater are given in Table S1.

Table S1. Conditions for the spin-coating method.

Step	Time (s)	Speed (rpm)	Acceleration (rpm)
1	10	1000	
2	37	2500	1000
3	59.1	2000	

S2. Carbon Capture Absorption Experiment

To measure CO_2 absorption, we used both dynamic and static methods. The dynamic method was adopted from a previous report,[3] where a high-pressure chamber equipped with a quartz crystal microbalance (QCM) was used to measure gas sorption within the film through the gravimetric measurement. The static method is a variation of pressure drop approach.[4,5] Because obtaining reliable data for IL, via dynamic approach, was challenging, we only relied on the result from the static method and compared the results against the reported data in the literature.[6] These data are shown in Table S2.

S2.1. Dynamic Method

S2.1.1. Experimental Apparatus

Figure S2 illustrates a schematic representation of the experimental apparatus used measuring CO_2 sorption. As shown in Figure S2, a QCM flange is connected to a 4-way Standard Cross purchased, serving as a pressure chamber, and isolated from the gas tank by a pressure regulator (Equilibar Inc., Fletcher, NC, USA, EPR 500). A pressure readout (MKS, Andover, MA, USA, Baratron Pressure Transducer, 870B-24539), attached to the cross, is used to read the overhead pressure of the QCM module. The chamber is evacuated using a rotary vane pump (Edwards, West Sanborn, NY, USA, RV5 One-Stage), and in inline valve was used for isolation during sorption measurement. The pressure readout data is transferred in real-time to a desktop via a microcontroller (Arduino, Somerville, MA, USA, Uno Rev 3), and the data is logged into LabVIEW software. To record the frequency and temperature of the QCM, the flange is connected to the Eon-LT sensor (not shown in Figure S2), and data are logged. To maintain the temperature of QCM at its set point, a recirculating chiller (PolyScience, Niles, IL, USA, AD15R-30-A11B) is used.

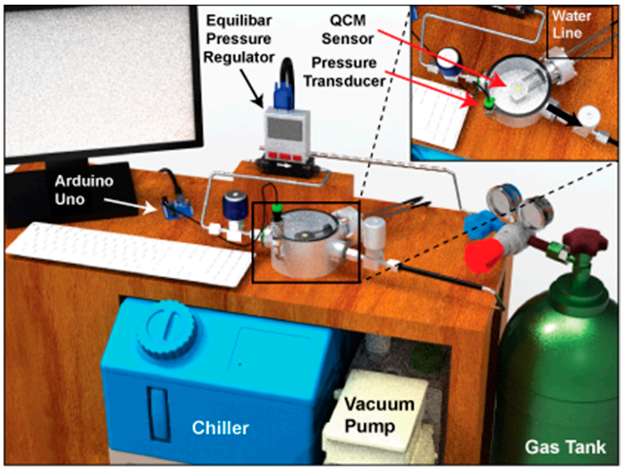

Figure S2. A schematic of an experimental apparatus for the dynamic method. The QCM flange shown in this figure only displays for the representative. Please refer to an experimental apparatus for an accurate dimension.

S2.1.2. Experimental Analysis

To estimate the adsorbed mass of CO_2 within thin films Equation (S1) was used.

$$\frac{m_{CO_2}}{m_{film}} = \frac{\left[\left(f_{film,Pi} - f_{film,Po}\right) - \left(f_{QC,Pi} - f_{QC,Po}\right)\right]}{\left(f_{film,Po} - f_{QC,Po}\right)} \quad \text{(S1)}$$

where:

m_{CO2}—Adsorbed mass of Carbon Dioxide (mg)
m_{film}—Mass of coated film (mg)
$f_{film,Pi}$—Frequency of coated film exposed at pressure, P_i (Hz)
$f_{film,Po}$—Frequency of coated film in vacuum, P_0 (Hz)
$f_{QC,Pi}$—Frequency of bare QC exposed at pressure, P_i (Hz)
$f_{QC,Po}$—Frequency of bare QC in vacuum, P_0 (Hz)

Figure S3 illustrates one typical pressure and frequency trajectory collected for both bare and coated QCM at three different pressures—100, 150, and 200 psi. Here for each experiment, we recorded the frequency and pressure traces at four different temperatures of 10, 20, 30, and 40 °C and eight different pressures of 50, 100, 125, 150, 175, 200, 225, 250 psi.

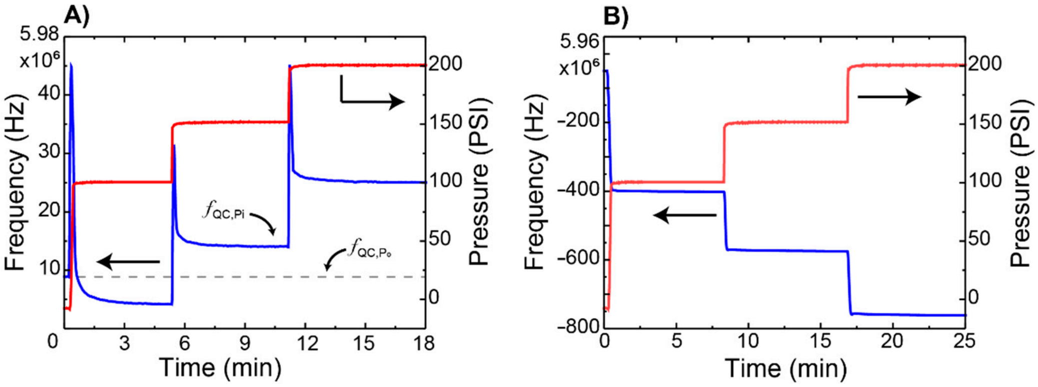

Figure S3. Typical pressure and frequency traces, collected from the QCM module. Here the data is collected at 10 °C and the pressure set points were 100,150, 200 psi. (**A**) Bare and (**B**) coated QC with 50:50 wt % PVDF-HFP:IL.

S2.2. Static Gravimetric Method

S2.2.1. Experimental Apparatus

Figure S4a illustrates an experimental apparatus used for the static gravimetric method in this study. As shown in Figure S4a, the main cell, which contains adsorbents, is attached to a manifold, connected to a gas tank through the pressure regulator. A pressure transducer (Honeywell, Charlotte, NC, USA, PX2AN1XX200PSAAX) is connected to the cell, and the pressure data is logged into LabVIEW. The main cell (except for a section connected the pressure read-out) is emerged in the chiller (PolyScience, Niles, IL, USA, AD15R-30-A11B) for maintaining a constant temperature.

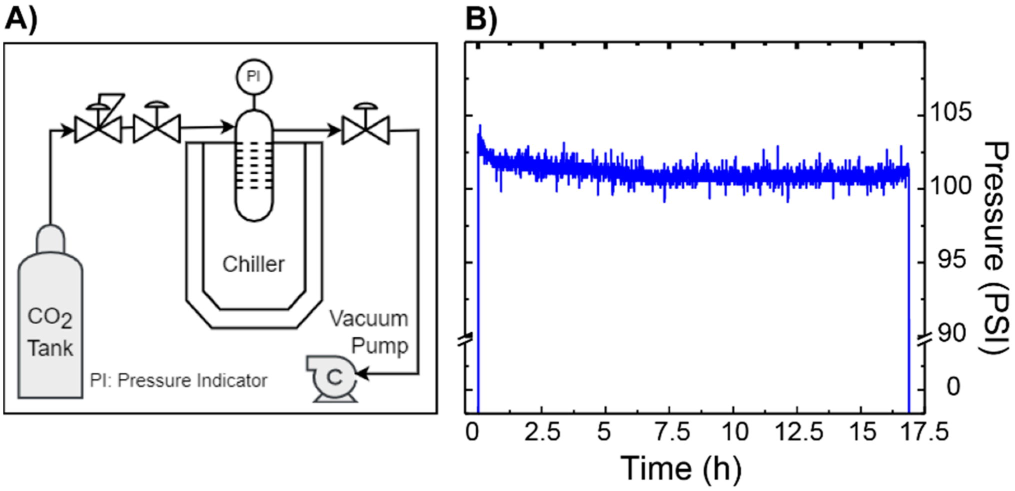

Figure S4. (**A**) A schematic of the gas manifold and the component of the apparatus used for measurement using static method. (**B**) Typical pressure trajectory gathered from the cell. The pressure approached a plateau after ~10 h.

S2.2.2. Experimental Method

Before testing, the cell (Swagelok, La Vista, NE, USA, 316-SS Welded VCR Face Seal Fitting, 1/4 in. Rotating Female Union) and a cell's cap (Swagelok, La Vista, NE, USA, 316-SS VCR Face Seal Fitting, 1/4 in. Plug) were cleaned with soap followed by acetone; then, they were transferred into a beaker of 250-mL acetone and sonicated for 10 min followed by being heated in an oven at 95 °C for 4 h and cooled down to the room temperature. A clean and dry cell, capped on 1-end, was placed on an analytical balance (Sartorius, Bohemia, USA, MSA225P100DI Cubis Analytical Balance) and tared. After the weight was initialized, IL solution was pipetted into the cell using a glass disposable pipette tip, and the weight was recorded. Then, the cell was sealed on the main manifold as shown in Figure S4. The whole system was evacuated, and the temperature on the circulating bath was set. Initially, the system was heated at 75–76 °C for ~ 10 h continuously, while being evacuated, then it was cooled down to the desired temperature and allowed to reach equilibrium (~ 8 h). Before introducing CO_2 to the system, the vacuum-line valve was closed, and the pressure logger was initiated. When the desired pressure was reached, the inlet valve was closed; when the pressure reached a plateau, the test was stopped. For the regeneration process, the vacuum pump was turned on, and the vacuum-line valve was gradually opened to evacuate the system. Figure S4b illustrates the typical pressure trajectory from the transducer.

S2.2.3. Absorption from static cell

To calculate adsorbed mass of CO_2, the Peng-Robinson Equation of State (PR-EOS) is used, and it can be shown in Equation (S2):

$$P = \frac{RT}{(V-b)} - \frac{a\alpha}{[V(V+b)+b(V-b)]} \quad \text{(S2)}$$

$$a = \frac{0.45724 \times \mathrm{R}^2 \times (Tc)^2}{Pc}$$

$$b = \frac{0.07780\mathrm{R} \times \mathrm{Tc}}{Pc}$$

$$a = [1 + (0.37464 + 1.54226\omega - 0.2699\omega^2) \times (1 - (Tr)^2)]^2$$

Whereas P and T are the pressure of the adsorbate at equilibrium (psi) and the absolute temperature (K), respectively; R is the ideal gas constant (8.314 $\mathrm{Jmol^{-1}K^{-1}}$); V is the volume of the cell (mL); ω is the acentric factor of CO_2; Tc and Pc is the critical pressure of CO_2.

Equation (S2) is then re-stated into polynomial form, which is shown in Equation (S3):

$$Z^3 - (1-B)Z^2 + (A - 2B - 3B^2)Z - (AB - B^2 - B^3) = 0 \quad \text{(S3)}$$

$$A = 0.45724\mathrm{a} \times \mathrm{P}_r \times (\mathrm{T}_r)^{-2}$$

$$\mathrm{B} = 0.45724\mathrm{a} \times \mathrm{P}_r \times \mathrm{T}_r$$

$$Z = \frac{PV}{nRT}$$

Whereas P_r is the reduced pressure; T_r is the reduced temperature; Z is the compressibility factor, which is calculated by applying Newton-Raphson method to solve the cubic equation. With Equation (S2) a-b mentioned above, the final value is computed by using MATLAB software.

S2.3. Sorption Results

S2.3.1. Static Method

Table S2. The static gravimetric isotherm sorption results of [EMIM][TF_2N] (Mole CO_2 per kg IL) at 4 different pressures (psi) and temperatures (K). At 100 and 150 psi, an average and standard deviation were reported based on 3 different prepared tests. At 200 psi, an average and standard deviation were reported based on 2 different prepared tests. Data is compared with interpolated reported values.[6–9].

**T (K)**	**This work**				**Report** [6–9]			
	283.15	293.15	303.15	313.15	283.15	293.15	303.15	313.15
**P (psi)**								
100	0.91 ± 0.08	0.65 ± 0.01	0.57 ± 0.02	0.55 ± 0.01	0.73	0.86	0.67	0.49
150	1.16 ± 0.04	0.89 ± 0.06	0.77 ± 0.04	0.73 ± 0.05	1.06	1.13	0.88	0.66
200	1.28 ± 0.08	1.19 ± 0.01	1.06 ± 0.04	0.98 ± 0.02	1.32	1.44	1.11	0.85

S2.3.2. Dynamic Method

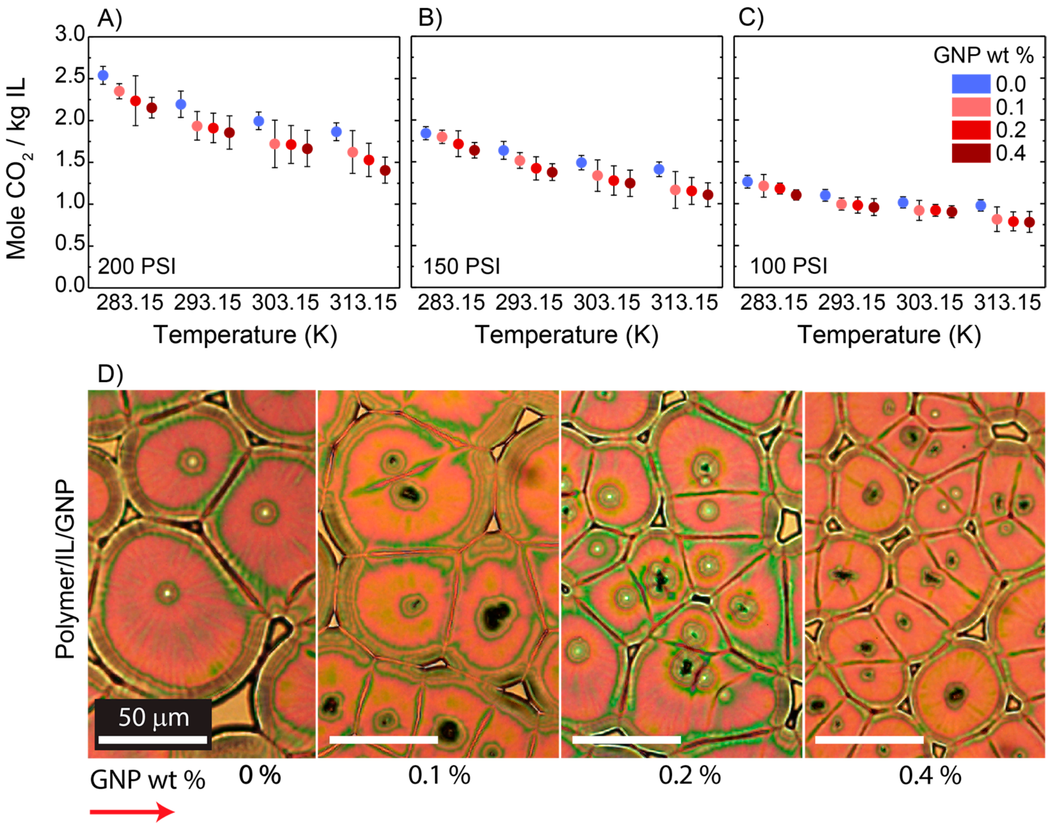

Figure S5. The effect of adding IL on the equilibrium absorption capacity of PVDF-HFP:IL (GNP) mixtures at three different pressures of (**A**) 100, (**B**) 150, and (**C**) 200 psia. Each data point is the average of four measurements presented with a standard deviation. (**D**) Polarized Microscopy Images showing the spherulites grown from different Polymer/IL mixtures with various GNP content. A scalebar is applicable for all subfigures D.

S2.3.3. Heat of Absorption

The enthalpy (or heat) of absorption was estimated by conducting separate experiments, where the temperature were kept constant, and the equilibrium pressures and the corresponding mole uptake were measured. From the slopes of the graph of the natural logarithm of dimensionless pressure vs. inverse temperature (in Kelvin), the heat of absorption under isosteric condition were estimated following Equation (S4) [10]

$$-\frac{\Delta H_{ads}}{r} = \frac{d}{dT}\left[\ln\left(\frac{P}{kPa}\right)\right]_N \quad \text{(S4)}$$

Where P and T are the pressure of the adsorbate at equilibrium (kPa) and the absolute temperature (K), respectively; R is an ideal gas constant (R = 8.314 x 10^{-3} kJmol^{-1}K^{-1}); N is the excess molar concentration; ΔH_{ads} is an enthalpy of sorption (kJ/mol). An example of the enthalpy of adsorption is shown in Tables S3 & S4.

Table S3. Sorption isotherms dataset of 50:50 Polymer:IL at different temperatures and 11 different pressures. The data are the average of five different measurements presented with one standard deviation.

P		Temperature (K)			
		283.66 ± 0.2	293.81 ± 0.1	303.25 ± 0.2	312.81 ± 0.3
(psi)	(kPa)	Mole CO_2 / kg IL			
50	344.7	0.631 ± 0.02	0.593 ± 0.05	0.520 ± 0.06	0.528 ± 0.07
75	517.1	0.899 ± 0.04	0.819 ± 0.05	0.761 ± 0.06	0.748 ± 0.07
100	689.5	1.215 ± 0.02	1.094 ± 0.07	1.010 ± 0.07	0.974 ± 0.07
125	861.8	1.530 ± 0.02	1.385 ± 0.05	1.252 ± 0.08	1.193 ± 0.08
150	1034.2	1.842 ± 0.03	1.638 ± 0.11	1.490 ± 0.09	1.411 ± 0.09
175	1206.6	2.189 ± 0.06	1.936 ± 0.10	1.743 ± 0.09	1.637 ± 0.11
200	1378.9	2.540 ± 0.10	2.195 ± 0.16	1.996 ± 0.11	1.867 ± 0.11
225	1551.3	2.823 ± 0.04	2.447 ± 0.11	2.240 ± 0.12	2.088 ± 0.12
250	1723.7	3.138 ± 0.04	2.711 ± 0.12	2.488 ± 0.13	2.313 ± 0.13
275	1896.1	3.453 ± 0.04	3.021 ± 0.16	2.741 ± 0.11	2.580 ± 0.14
300	2068.4	3.769 ± 0.04	3.289 ± 0.17	2.977 ± 0.12	2.798 ± 0.15

Table S4. The enthalpy of adsorption of 50:50 Polymer:IL at different temperatures and 1.75 mole CO_2 / kg IL. The data are the average of five different measurements presented with one standard deviation.

T (K)	E^3/T	P (kPa) at 1.75 mole CO_2 / kg IL	ln(P/kPa)	Slope $-\Delta H_{ads}/R$	$-\Delta H_{ads}$ (kJ/mol)
283.66 ± 0.2	3.53	971 ± 18	6.88 ± 0.03	−821.9 ± 172	6.8 ± 1
293.81 ± 0.1	3.40	1072 ± 45	6.98 ± 0.07		
303.25 ± 0.2	3.30	1185 ± 56	7.08 ± 0.08		
312.81 ± 0.3	3.20	1268 ± 61	7.14 ± 0.08		

R^2—value (for the linear fitting of the slope) = 0.976 ± 0.006.

Similarly, the enthalpy of CO_2 absorption in polymeric mixtures with various content of IL and GNP were determined; the data are presented in Figure S6.

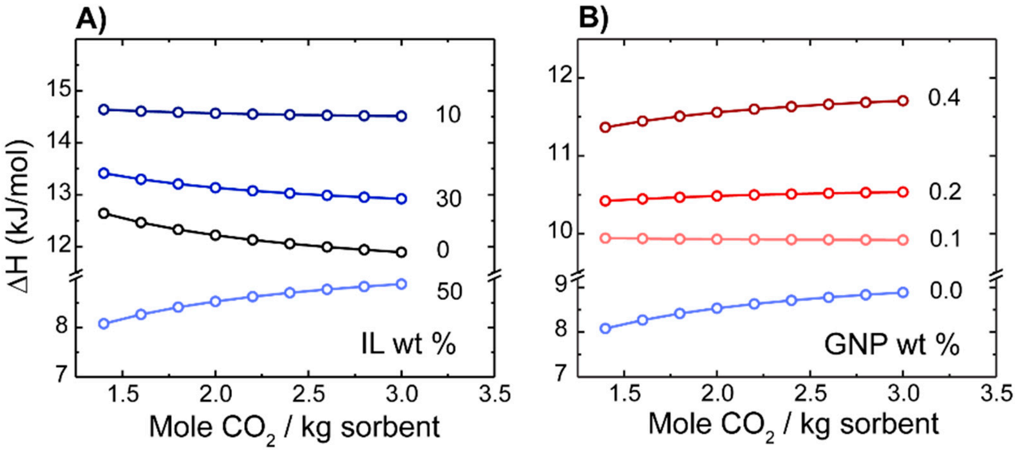

Figure S6. Enthalpy of adsorption of polymeric mixtures. (**A**) PVDF-HFP:IL with various concentrations of IL. (**B**) Polymeric mixtures of 50:50 PVDF-HFP:IL with various concentrations of GNP.

To gain quantitative information on the interaction between CO_2 and the coated-thin film of Polymer:IL (GNP), the enthalpy of adsorption of CO_2 for polymeric films with various concentrations of IL and GNP are compared. As shown in Figure S6a, at the

higher mole uptake region, the enthalpy of absorption for CO_2 in neat polymer and samples containing up to 30 wt % IL were decreased. The trend is reversed for higher content of ILs. At constant excess molar concentration, we can see that the enthalpy of sorption as a function of IL content initially increases and then decreases. We attribute the reduction in the enthalpy of sorption to the enhanced mobility of chain, or a better dispersion of IL within polymer domain. As the GNP added to the mixture the interaction of the Polymer:IL is against weakened due to the role of GNP as a nucleation agent.[11,12]

S3. Film Characterization

S3.1. Atomic Force Microscopy (AFM)

S.3.1.1. Sample Preparation

For AFM analysis, thin films were prepared by spin coating of solutions on polished silicon wafers and dried using the same processing conditions as noted before. The AFM measurements were conducted using a Bruker Dimension Icon AFM in tapping mode with a range of 15 µm. The ScanAsyst-Air tip was used in this study.

S.3.1.2. Additional AFM images

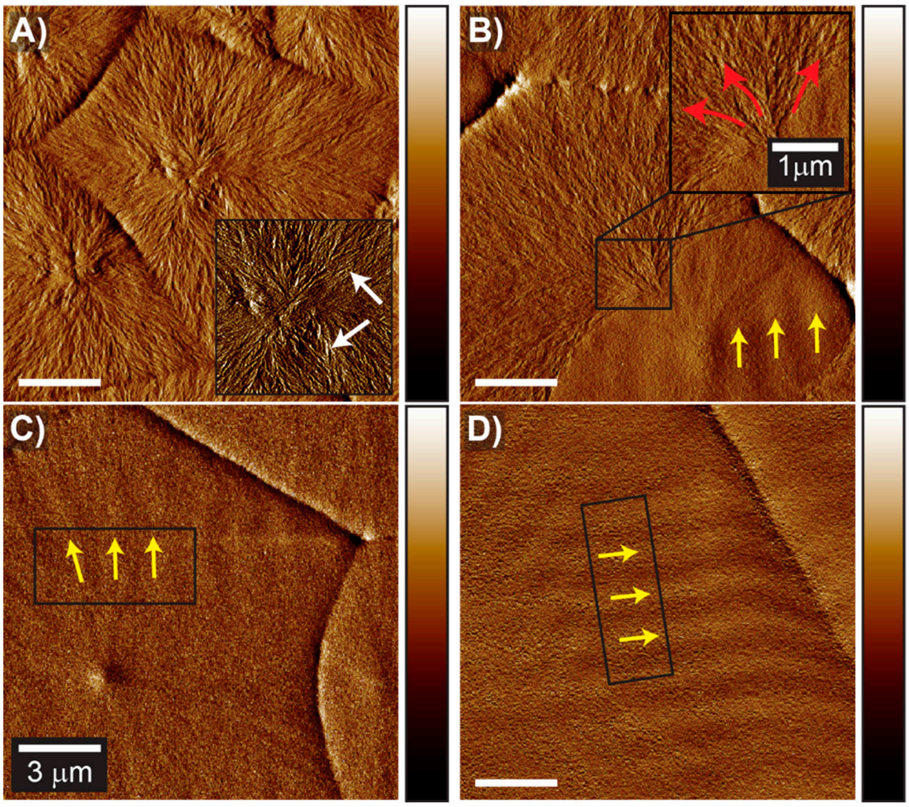

Figure S7. AFM inphase images of the sample spin-cast from different solutions of Polymer:IL as specified in the figure: (**A**) 0 wt. % IL, (**B**) 10 wt. % IL, (**C**) 30 wt. % IL, (**D**) 50 wt. % IL. Arrows indicates the lamella direction: a yellow arrow indicates the orientation of fibrils of the edge-on lamellae, while a red arrow indicate the flat-on filbrils; a white arrow indicates the small fibrils. A scalebar is applicable to all figures.

S3.2. Polarized Microscopy (PLM) and Spherulite Growth Rate

Polarized microscopy measurements were conducted using an Olympus BX51 Polarizing Microscope equipped with a Mettler Toledo FP900 thermal system with a temperature range from room temperature to 375 °C. Thin films were prepared on polished silicon wafers.

S3.2.1. Polarized Microscopy

The size of spherulite and the number of nuclei were analyzed using the FIJI (ImageJ2) software.[13] For the PLM of the samples containing 50 wt % ILs, the Trainable Weka Segmentation (TWS) method,[14] was applied to estimate the size and number of spherulites. A typical image analysis using TWS method is illustrated in Figure S8. For the rest of the samples the measurements were done manually.

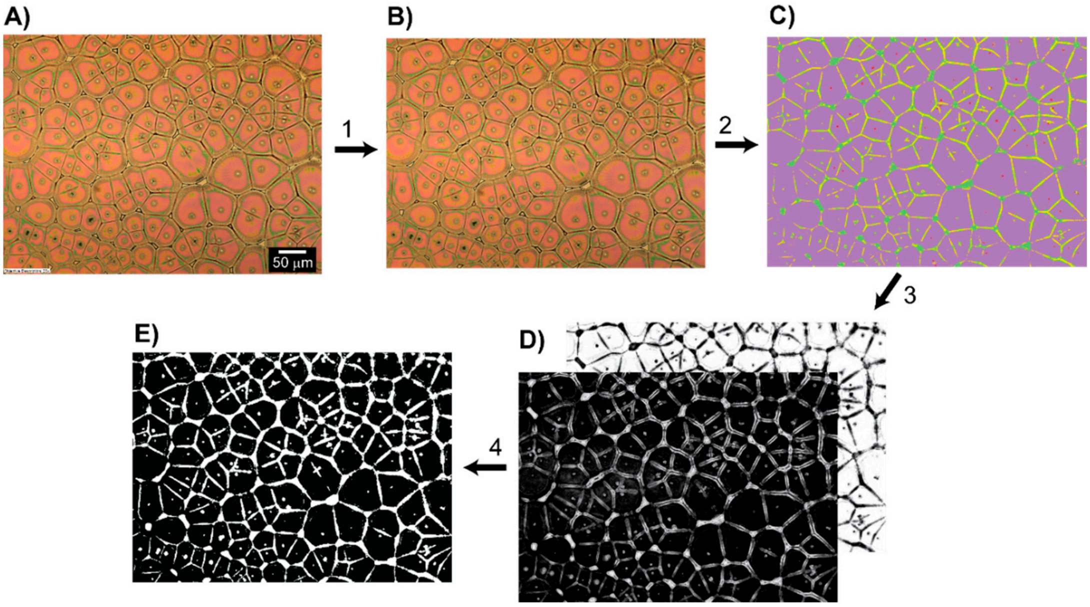

Figure S8. An overview PLM analysis is performed in 4 steps: (1) pre-processing image by scaling and selecting an image region; (2) an image classifier model is applied to obtain a resulted image from the "Trainable Weka Segmentation"; (3) an image threshold process is applied to a selected class based on users' defined classes; (4) binary convert process is applied to separate segmented regions whereas white color corresponds to a selected region. (**A**) an original PLM image. (**B**) a scaled image. (**C**) an image after the training was completed. (**D**) trained images with separated labels were created. (**E**) a binary image for post-processing.

S3.2.2. Spherulite Growth Rate Analysis

The spherulite growth were observed under polarized microscope. Thin films were coated on silicon wafers from the polymer solutions heated at 90°C. For quantitative analysis of spherulite growth rate (G), Heat Stage at a heating rate of 2 °C/min was used. The spherulite growth evolution was captured with a speed of 50 frame-per-second (fps). The first two spherulites that appeared in an image were selected as the representative spherulites. At each time frame, the radius of the selected spherulites was measured using FIJI. An example of a growth evolution for a spherulite is shown in Figure S9.

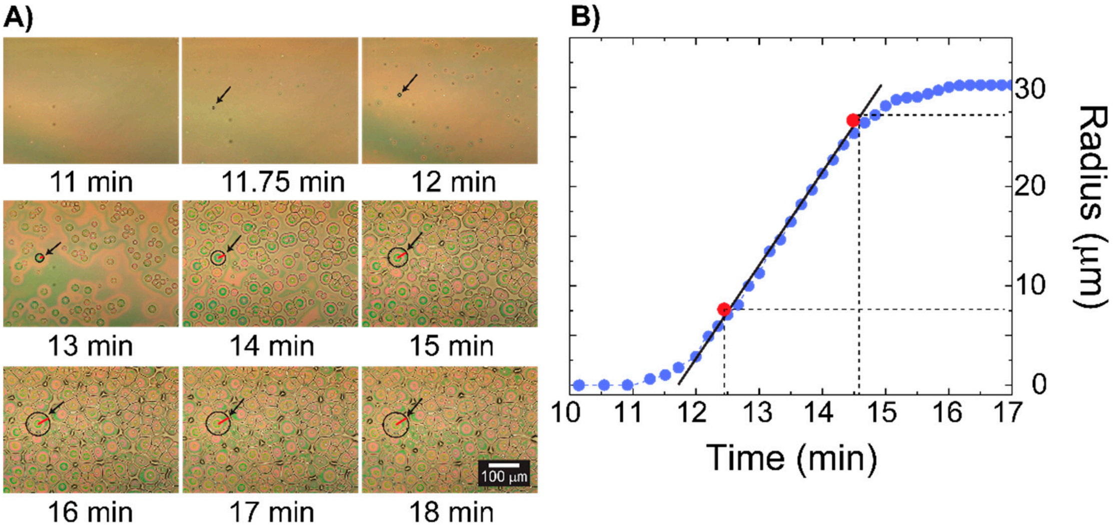

Figure S9. (**A**) Evolution of spherulites from a thin film of 50:50 PVDF-HFP:IL coated on a silicon wafer and heated with a heating rate of 2 °C/min; the red line indicates the radius of the selected spherulite. All figures have the same scale. (**B**) Spherulite growth rate curve. Each data point shown on the curve is the average value of two spherulites' radii.

The spherulite growth rate consists of different sub-processes, e.g., solvent evaporation, nucleation, crystal growth, and particle suspension. In this study, we only focused on the nucleation and the crystal growth. A growth rate (µm/min) is determined from the slopes of radius vs. time curves, and the values are listed in Table S5.

Table S5. Spherulite growth rate of polymeric mixture with various IL and GNP contents. The average and standard deviation of all samples were reported from two spherulites that appeared first on the surface.

IL wt % (GNP wt %)	Growth Rate (µm/min) (mean ± std.)
10	33.1 ± 2.9
30	15.4 ± 1.4
50	9.4 ± 0.04
50 (0.1)	9.2 ± 0.6
50 (0.2)	6.6 ± 1.8
50 (0.4)	6.4 ± 1.3

Std.— data is reported with one standard deviation.

S3.3. Fourier-transform infrared spectroscopy (FTIR)

To gain information about the film composition, FTIR measurements were performed on a Bruker ALPHA FTIR spectrometer. Thin film samples were prepared on the glass slide (VWR Precleaned Microscope Slides, 2.5x7.5x0.1cm) and were lift off the glass and placed on the diamond window of the spectrometer. The IR spectra were recorded in the ATR mode and used for further analysis. The IR peak assignments of PVDF-HFP, IL, and TEP are tabulated in Table S6. The spectra of thin films were used to study the composition of these films. To quantify the degree of β-phase, the frequency regions

from 1100 to 1300 cm⁻¹ containing both of α-, β-, ɣ-phase are chosen.[15] The selected region is shown in Figure S10.

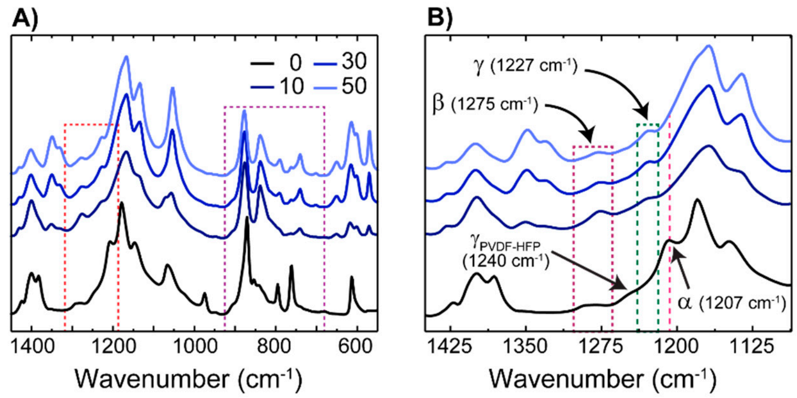

Figure S10. (**A**) FTIR spectra of polymeric mixtures; the red box indicates the frequency region, containing α-, β-, and ɣ-phase, which is used to determine the formation of ɣ-phase for the quantification of the degree of β-phase, the purple box indicates the frequency region used for the quantification of the degree of β-phase. (**B**) region in 1100–1450 cm⁻¹, containing α-, β-, ɣ-phase.

For PVDF-HFP, as shown in Figure S10b, β- and ɣ-phase appeared at 1275 and 1240 cm⁻¹, respectively; a shift for peaks assigned to the β-phase as the function of IL content can be noted.[16] The degree of β-phase was determined by applying Equation 5, following previous reports and ignoring the ɣ-phase as it is often reported to be very small compared to the other phases.[17]

$$F(\beta) = \frac{A_\beta}{\left(\frac{K_\beta}{K_\alpha}\right) A_\alpha + A_\beta} = \frac{A_\beta}{1.26 A_\alpha + A_\beta} \quad \text{(S5)}$$

Whereas A_α and A_β are the absorbencies at the wavenumbers of 763 cm⁻¹ and 840 cm⁻¹, respectively. K_α and K_β are the absorption coefficients at the respective wavenumbers with the values given as 6.1 × 10⁴ cm²/mol and 7.7 × 10⁴ cm²/mol, respectively.[17] Using Equation 5 along with the peak intensities, estimated from the deconvolution of spectra in between 790–811 cm⁻¹ (Table S7), was used to estimate the degree of β-phases in the samples. Table S8 presents the estimated degree of β-phase for films prepared from seven different mixtures.

Table S6. Characteristic FTIR peaks of polymeric film.

Wave number (cm^{-1})	Associated Peak Name	Sample	References
569	CF_3	[EMIM][TF_2N]	[2]
600	SO_2		
650	SNS		
741	CF_3		
1051	NCH_3		
1132	SO_2		
1348	SO_2		
1141	CF_3	PVDF-HFP	[18–21]
1641	CH=CF		
2900–3000	CH (sp^2-sp^3)		
1289	CF		
612, 1207	α-phase		
875, 853, 1275	β-phase		
836	β-phase (Polymer:IL films)		
927	P-O-P	TEP	[22]
1061	P-O		
1209	P=O		

Table S7. Defined peak locations for the peak convolution.

Peak number	Wavenumber (cm^{-1}) [used for PVDF-HFP:IL (GNP)]	Wavenumber (cm^{-1}) [used for PVDF-HFP]
1	702	726
2	725	738
3	740	761
4	750	795
5	762	805
6	790	842
7	811	854
8	839	871
9	877	885
10	904	907

Table S8. Relative fraction of β-phase of polymeric films. An asterisk (*) indicates that a value is estimated from an interpolation. Data is reported with one standard deviation.

IL wt % (GNP wt %)	Actual IL wt % (# prep. solutions) (mean ± std.)	F(β) (This work) (mean ± std.)	F(β) (reported) [23]
0	0 (1)	0.19 ± 0.08	0.12
10	10.2 ± 1.3E^{-5} (2)	0.89 ± 0.004	0.90
30	31.5 ± 0.01 (2)	0.78 ± 0.01	0.75
50	50 ± 0 (1)	0.64 ± 0.08	0.65 *
50 (0.1)	51.1 ± 0 (1)	0.72 ± 0.14	-
50 (0.2)	50.2 ± 0 (1)	0.74 ± 0.09	-
50 (0.4)	50.1 ± 1.3E^{-4} (2)	0.77 ± 0.07	-

By comparing results shown in Table S8 with the reported values,[23] we note an agreement between the two sets. From Table S8, the β-phase content unexpectedly increased when IL content was 10 wt %. This behavior is associated with the electrostatic interaction between the CF_2 group of PVDF-HFP and the cation group of IL, promoting the formation of the β-phase.[24] However, as the IL content increased in polymeric mixtures, the plasticization effect reduces these interactions, leading to a decrease in the fraction of β-phase.[23] In contrast, when GNP was added to the mixture, the β-phase fraction was increased again. Here, we think that GNP restrained the plasticization effect of IL, favored the formation of β-phase.[25,26]

S3.4. X-ray Diffraction (XRD)

For the XRD measurements, the thick film preparation method was applied. The XRD measurements were performed on Rigaku SmartLab (Rigaku Co., Tokyo, Japan, SmartLab). The source was Cu-Kα 0.154 nm; the Bragg-Brentano method and a normal detector were utilized. XRD angles were collected from 5 to 50 degrees with a scanning rate of 1 min/deg and a step-size of 0.1 degrees. For determining the degree of crystallinity, the AMORPH software was used.[27] The input parameters of the AMORPH is presented in Table S9.

The XRD measurements were repeated at least three times, and the mean values were reported. For the polymeric films with 30 wt % of IL, the test was done once. The results were compared against previous report to determine the reliability of our analysis.[23] Figure S11 illustrates typical XRD analysis, done with the AMORPH software; data for seven samples of different compositions are shown. The Miller indices and the diffraction angles of the major peaks are tabulated in Table S10.

Table S9. Parameters applied to AMORPH simulation to calculate the crystallinity and amorphousity of the polymeric film.[27].

Parameter	Value
Number of particles	1
New level interval	10,000
Save interval	25,000
Thread Steps (ThreadSteps)	100
Maximum number of levels	0
Backtracking scale length	10
Strength of effect to force histogram to equal push	100
Maximum number of saves	10,000
Control points	[5.0–10.0,50.0]
Number of threads (num_threads)	30–34
Subset of the domain should the amorphous peaks be allowed to appear in on the left edge	Vary (based on user's decision)
Subset of the domain should the amorphous peaks be allowed to appear in on the right edge	Vary (based on user's decision)

Table S10. Pattern XRD angles of the polymeric film.[28–30].

Peak's Type	α	α	$\beta_{PVDF-HFP}$ (PVDF-HFP)	$\beta_{PVDF-HFP}$ (Polymer:IL films)	α	α	GNP	α
Planes	(100)	(020)	(200)	(200)	(200)	(002)	(002)	(021)
$2\theta°$	17.9	18.4	19	20	25	27	27.5	37

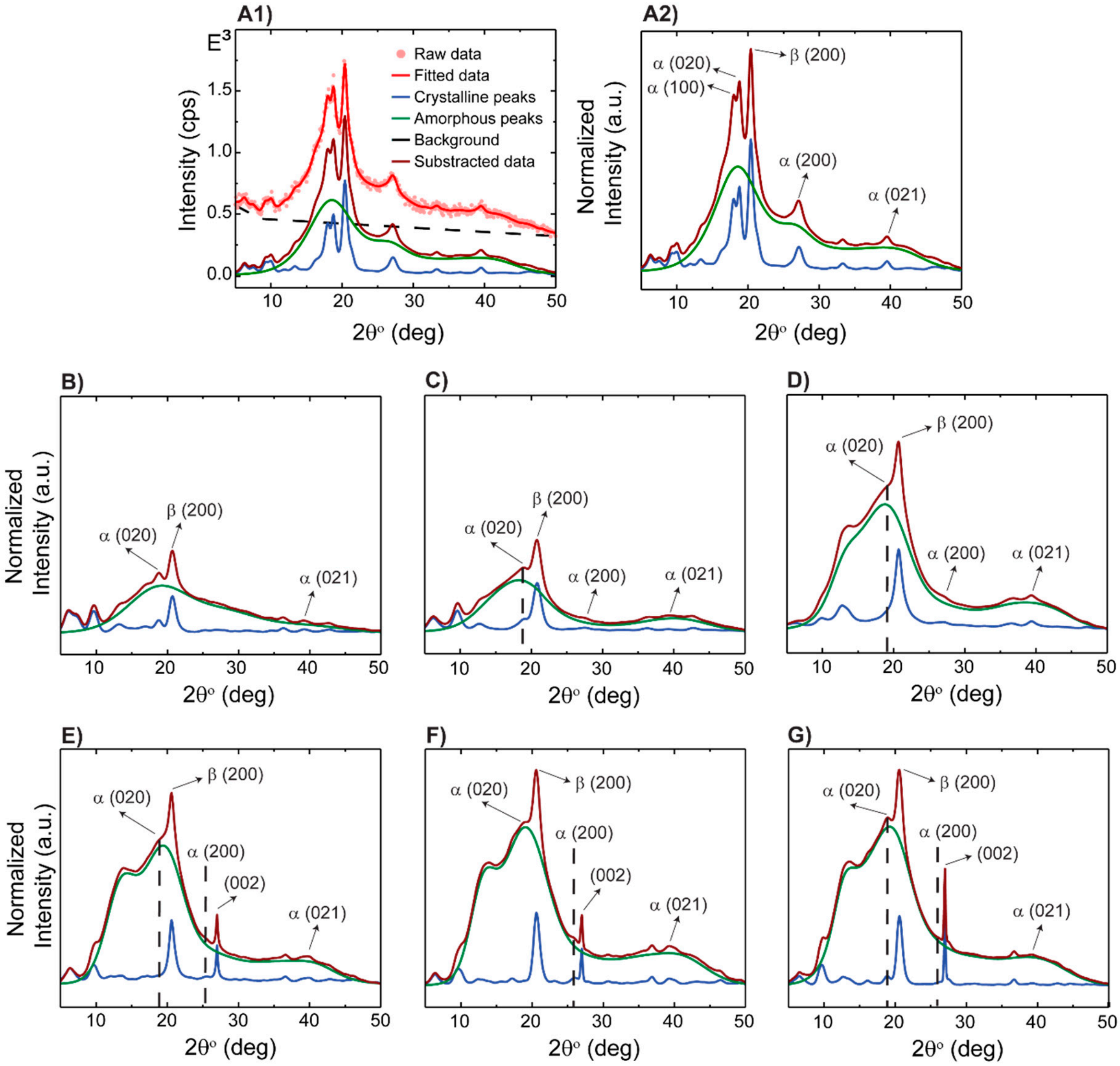

Figure S11. XRD Analysis of polymeric mixtures with various concentrations of IL and GNP. IL wt % in PVDF-HFP—(A) 0 wt %. (B) 10 wt %. (C) 30 wt %. (D) 50 wt %; GNP wt % in 50:50 PVDF-HFP:IL—(E) 0.1 wt %. (F) 0.2 wt %. (G) 0.4 wt %.

The amorph fractions (X_{amorph}) of polymeric films, prepared from various compositions of IL and GNP in polymer, were estimated from the output of software. The degree of crystallinity was calculated and tabulated in Table S11.

Table S11. The degree of crystallinity of polymeric films. For the films with 0, 10, and 50 wt % IL content. The "Half-width (modelling)" indicates the standard deviation of how close the model to an actual dataset is.[27].

IL wt % (GNP wt %)	Actual IL wt % (mean ± std.)	X_c % ± σ (%) (mean ± std.)	Half-width (modelling) (mean ± std.)
0	0	30.19 ± 0.11	9.23 ± 0.6
10	10.0 ± 0.07	22.25 ± 0.07	8.72 ± 0.6
30	32.1 ± 0.0	24.99 ± 0.12	9.79 ± 0.6
50	50.1 ± 0.1	19.83 ± 0.87	8.91 ± 1.3
50 (0.1)	49.9 ± 0.1	15.90 ± 0.14	8.86 ± 0.9
50 (0.2)	50.1 ± 0.07	10.86 ± 0.10	8.61 ± 0.5
50 (0.4)	50.2 ± 0.0	8.10 ± 0.05	9.41 ± 0.4

The result is in good agreement with previous report.[23] Figure S9 (first row), shows a diffractogram of PVDF-HFP films. The predominant peaks are located at 2θ equal to 18, 19, and 27 degrees corresponding to the α-phase (020), β-phase (200), and α-phase (200).[29,31] A small broad peak at 37 degrees is attributed to the 021 plane of the α-phase.[32] As can be seen, when IL is introduced to the polymeric films, the α-phase of PVDF-HFP is suppressed. This was evidenced by the reduction and the shift of two diffraction peaks at 2θ of 18 and 27 degrees, associated with the α-phase (020) and (200). These changes in the structure of polymer were attributed to the strong van der Waals (vdW) interaction between the imidazolium cations of IL and the negative dipoles of CF_2 groups of PVDF-HFP. These interactions appear to be the rate determining parameter of crystallization kinetics and stabilized the formation of the β-phase of PVDF-HFP.[33,34] Previous reports point to similar trends; here we think that the mobility of IL induces disorder in the arrangement of polymer chain, resulting in a reduction in the degree of crystallinity and the formation of the β-phase.[23,35]

S3.5. Differential Scanning Calorimetry (DSC)

Differential Scanning Calorimetry (DSC) measurements are used to determine the degree of crystallinity and melting temperature of semicrystalline polymer.[36] Thick film samples were prepared with the same composition as noted in the XRD section. The film were cut into small pieces, and ~ 5.5 mg was used for the measurement. The DSC measurements were performed on the Netzsch DSC 204 F1 Phoenix Differential Scanning Calorimeter at a heating/cooling rate of 5 °C/min under N_2 flow.

Figure S12 shows DSC curves of all samples for the heating process from 25 to 175 °C at 5 °C/min, and Figure S11 shows DSC curves for all samples for the cooling process from 175 °C to the room temperature at 5 °C/min. As shown in Figure S13, there were two peaks associated with two melting temperature (T_m), on the DSC curves, indicating two types of crystal phases—α- and β-phase.[24,37] Table S12 summarized the melting temperature for polymeric films, and Table S13 summarized the crystallization and solidification temperature for polymeric films. Compared with pure PVDF-HFP, as the IL content increased, T_m value decreased. The shift in T_m to the lower region was due to the plasticization effect of IL.[24] It can be also associated with the reduction in the crystallite size and the increase in interfacial area, which was previously reported.[38] When GNP was added to the polymeric films, because of the nucleation effect of GNP, the β-phase of PVDF-HFP was increased and the melting point of the film was further re-

duced. Furthermore, when the IL or GNP content was increased, the exothermic peaks became wider, indicating the slower crystallization rate and the lower degree of crystallinity of the films. This trend is consistent with the XRD measurements, spherulite growth rate analysis, and previous reports.[37,39]

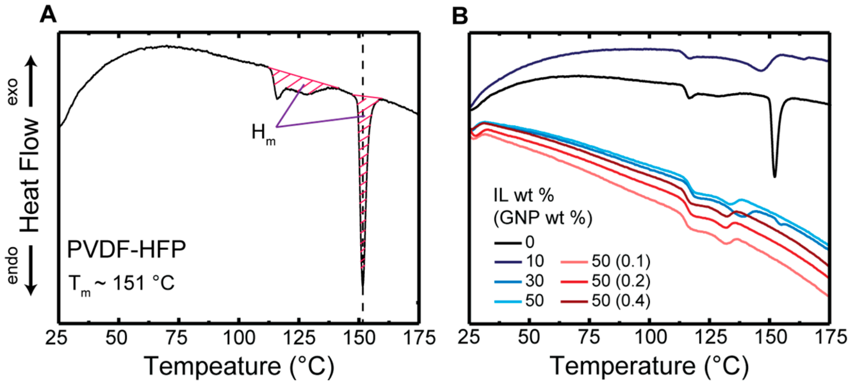

Figure S12. (A) DSC curve analysis of PVDF-HFP. (B) DSC curve of Polymer:IL (GNP).

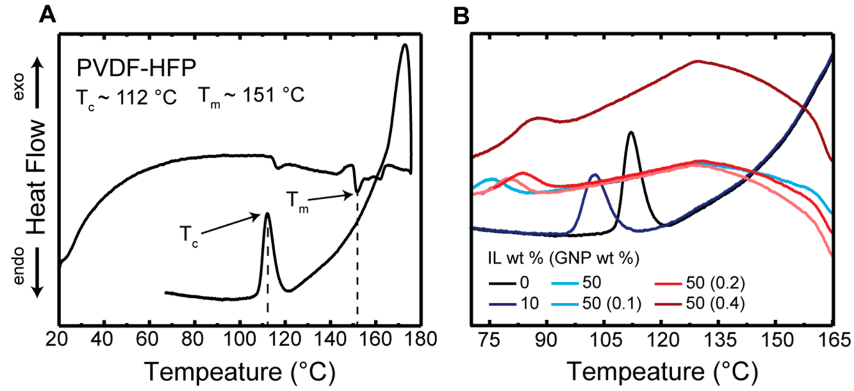

Figure S13. (A) Heat Flow vs. Temperature (°C) curve analysis of PVDF-HFP. (B) Heat Flow vs. Temperature (°C) curve of Polymer:IL (GNP) during a cooling process.

By assuming the film is mostly ordered into α- and β-phase, the degree of Crystallinity (X_c) was calculated using following Equation 7.[40]

$$X_c = \frac{\Delta H_m}{x \times \Delta H_\alpha + y \times \Delta H_\beta} = \frac{\Delta H_m}{(1-y) \times \Delta H_\alpha + y \times \Delta H_\beta} \quad (7)$$

Where ΔH_β is the heat of fusion of $\beta_{PVDF\text{-}HFP}$ (~ 104.7 J/g);[40] ΔH_α is the heat of fusion $\alpha_{PVDF\text{-}HFP}$ (~ 93.04 J/g);[40] x is a relative fraction of degree of α-phase of PVDF-HFP; y is a relative fraction of degree of β-phase of PVDF-HFP.

Table S12. Melting temperature of various films. For the films with 50 wt % IL content the data are representative of average values of three measurements from different samples, presented with one standard deviation.

IL wt % (GNP wt %)	Actual IL wt %	T_m (°C) (This work) (mean ± std.)	T_m (°C) (reported)	References
0	0	151.7 ± 0.1	144.9, 145, 147.2, 151	[39,41–44]
10	10.2	146.8	134	[23]
30	32.1	138.7 ± 0.4	129	[23]
50	50.2	132.2 ± 0.5	110	[23]
50 (0.1)	49.9	131.8 ± 0.4	-	-
50 (0.2)	50.0	131.6 ± 0.3	-	-
50 (0.4)	50.2	130.4 ± 2.6	-	-

Table S13. Melting temperatures, crystallization temperatures, and solidification of various films. For the films with 50 wt % IL content, the data are representative of average values of three different samples, presented with one standard deviation.

IL wt % (GNP wt %)	Actual IL wt %	T_m (°C) (mean ± std.)	T_c (°C) (mean ± std.)	T_c (°C) (reported)	Solidification (°C) ($\Delta T = T_m - T_c$)
0	0	151.7 ± 0.1	110.8	112.5 [44]	40.9 ± 1.5
10	10.2	146.8	103.1	-	43.8
30	32.1	138.7 ± 0.4	-	-	-
50	50.2	132.2 ± 0.5	128.8 ± 0.8	-	3.4 ± 0.9
50 (0.1)	49.9	131.8 ± 0.4	130.1 ± 0.6	-	1.8 ± 0.7
50 (0.2)	50.0	131.6 ± 0.3	130.9 ± 0.5	-	0.7 ± 0.5
50 (0.4)	50.2	130.4 ± 2.6	129.9 ± 0.2	-	0.6 ± 2.6

Since $X_c = \Delta H_m / (x^*\Delta H_\alpha + y^*\Delta H_\beta) = \Delta H_m / ((1-y)^*\Delta H_\alpha + y^*\Delta H_\beta)$, the standard deviation of degree of crystallinity used for the DSC measurements can be determined by Equation 8.

$$\delta_{\chi_c} = \sqrt{\left(\frac{\partial \chi_c}{\partial \Delta H_m}\right)^2 s_{\Delta H_m}^2 + \left(\frac{\partial \chi_c}{\partial \mathrm{y}}\right)^2 s_{\mathrm{y}}^2} \quad (8)$$

Whereas

$$\frac{\partial \chi_c}{\partial \Delta H_m} = \frac{1}{(1-y) \times \Delta H_\alpha + y \times \Delta H_\beta}$$

$$\frac{\partial \chi_c}{\partial \mathrm{y}} = \frac{\Delta H_m (\Delta H_\alpha - \Delta H_\beta)}{[(1-y) \times \Delta H_\alpha + y \times \Delta H_\beta]^2}$$

Table S14. Degree of crystallinity of Polymer:IL (GNP).

IL wt % (GNP wt %)	$X_c \pm \sigma$ x 10^{-3} (%) (DSC, this work) (mean ± std.)	$X_c \pm \sigma$ x 10^{-3} (%) (XRD, this work) (mean ± std.)	X_c (%) (reported)
0	20.5 ± 34	28.29 ± 0.12	22.5,[37] 23,[42] 21[32]
10	17.9	22.25 ± 0.07	20 [23]
30	14.6 ± 0.4	24.99 ± 0.12	22.5 [23]
50	10.8 ± 8	19.83 ± 0.87	19 [23]
50 (0.1)	10.4 ± 9	15.90 ± 0.14	-
50 (0.2)	10.2 ± 3	10.86 ± 0.10	-
50 (0.4)	9.6 ± 2	8.10 ± 0.05	-

Although the absolute value of crystallinity estimated from DSC and XRD are not in the same range, both the data set present the same trend. In the case of GNP-containing films, the estimated values for the crystallinity measured from DSC and XRD are similar.

References

1. Manna, S.; Batabyal, S. K.; Nandi, A. K. Preparation and Characterization of Silver−Poly(Vinylidene Fluoride) Nanocomposites: Formation of Piezoelectric Polymorph of Poly(Vinylidene Fluoride). *J. Phys. Chem. B* **2006**, *110* (25), 12318–12326. https://doi.org/10.1021/jp061445y.
2. Höfft, O.; Bahr, S.; Kempter, V. Investigations with Infrared Spectroscopy on Films of the Ionic Liquid [EMIM]Tf2N. *Langmuir* **2008**, *24* (20), 11562–11566. https://doi.org/10.1021/la802045j.
3. Baltus, R. E.; Culbertson, B. H.; Dai, S.; Luo, H.; DePaoli, D. W. Low-Pressure Solubility of Carbon Dioxide in Room-Temperature Ionic Liquids Measured with a Quartz Crystal Microbalance. *J. Phys. Chem. B* **2004**, *108* (2), 721–727. https://doi.org/10.1021/jp036051a.
4. Anthony, J. L.; Maginn, E. J.; Brennecke, J. F. Solubilities and Thermodynamic Properties of Gases in the Ionic Liquid 1-n-Butyl-3-Methylimidazolium Hexafluorophosphate. *J. Phys. Chem. B* **2002**, *106* (29), 7315–7320. https://doi.org/10.1021/jp020631a.
5. Shiflett, M. B.; Maginn, E. J. The Solubility of Gases in Ionic Liquids. *AIChE Journal* **2017**, *63* (11), 4722–4737. https://doi.org/10.1002/aic.15957.

6. Cadena, C.; Anthony, J. L.; Shah, J. K.; Morrow, T. I.; Brennecke, J. F.; Maginn, E. J. Why Is CO_2 So Soluble in Imidazolium-Based Ionic Liquids? *J. Am. Chem. Soc.* **2004**, *126* (16), 5300–5308. https://doi.org/10.1021/ja039615x.
7. Schilderman, A. M.; Raeissi, S.; Peters, C. J. Solubility of Carbon Dioxide in the Ionic Liquid 1-Ethyl-3-Methylimidazolium Bis(Trifluoromethylsulfonyl)Imide. *Fluid Phase Equilibria* **2007**, *260* (1), 19–22. https://doi.org/10.1016/j.fluid.2007.06.003.
8. Mirarab, M.; Sharifi, M.; Ghayyem, M. A.; Mirarab, F. Prediction of Solubility of CO_2 in Ethanol–[EMIM][Tf2N] Ionic Liquid Mixtures Using Artificial Neural Networks Based on Genetic Algorithm. *Fluid Phase Equilibria* **2014**, *371*, 6–14. https://doi.org/10.1016/j.fluid.2014.02.030.
9. Carvalho, P. J.; Álvarez, V. H.; Machado, J. J. B.; Pauly, J.; Daridon, J.-L.; Marrucho, I. M.; Aznar, M.; Coutinho, J. A. P. High Pressure Phase Behavior of Carbon Dioxide in 1-Alkyl-3-Methylimidazolium Bis(Trifluoromethylsulfonyl)Imide Ionic Liquids. *The Journal of Supercritical Fluids* **2009**, *48* (2), 99–107. https://doi.org/10.1016/j.supflu.2008.10.012.
10. Brunauer, S. Chapter VIII: The Heat of Adsorption II. In *The adsorption of gases and vapors*; H. Milford, Oxford University Press, 1943; Vol. 1, pp 218–270.
11. Zheng, X.; Sauer, B. B.; Van Alsten, J. G.; Schwarz, S. A.; Rafailovich, M. H.; Sokolov, J.; Rubinstein, M. Reptation Dynamics of a Polymer Melt near an Attractive Solid Interface. *Phys. Rev. Lett.* **1995**, *74* (3), 407–410. https://doi.org/10.1103/PhysRevLett.74.407.
12. Shih, C.-J.; Vijayaraghavan, A.; Krishnan, R.; Sharma, R.; Han, J.-H.; Ham, M.-H.; Jin, Z.; Lin, S.; Paulus, G. L. C.; Reuel, N. F.; Wang, Q. H.; Blankschtein, D.; Strano, M. S. Bi- and Trilayer Graphene Solutions. *Nature Nanotech* **2011**, *6* (7), 439–445. https://doi.org/10.1038/nnano.2011.94.
13. Schindelin, J.; Arganda-Carreras, I.; Frise, E.; Kaynig, V.; Longair, M.; Pietzsch, T.; Preibisch, S.; Rueden, C.; Saalfeld, S.; Schmid, B.; Tinevez, J.-Y.; White, D. J.; Hartenstein, V.; Eliceiri, K.; Tomancak, P.; Cardona, A. Fiji: An Open-Source Platform for Biological-Image Analysis. *Nat Methods* **2012**, *9* (7), 676–682. https://doi.org/10.1038/nmeth.2019.
14. Arganda-Carreras, I.; Kaynig, V.; Rueden, C.; Eliceiri, K. W.; Schindelin, J.; Cardona, A.; Sebastian Seung, H. Trainable Weka Segmentation: A Machine Learning Tool for Microscopy Pixel Classification. *Bioinformatics* **2017**, *33* (15), 2424–2426. https://doi.org/10.1093/bioinformatics/btx180.
15. Ghosh, S. K.; Alam, M. M.; Mandal, D. The in Situ Formation of Platinum Nanoparticles and Their Catalytic Role in Electroactive Phase Formation in Poly(Vinylidene Fluoride): A Simple Preparation of Multifunctional Poly(Vinylidene Fluoride) Films Doped with Platinum Nanoparticles. *RSC Adv.* **2014**, *4* (79), 41886–41894. https://doi.org/10.1039/C4RA06334A.
16. Ramasundaram, S.; Yoon, S.; Kim, K. J.; Park, C. Preferential Formation of Electroactive Crystalline Phases in Poly(Vinylidene Fluoride)/Organically Modified Silicate Nanocomposites. *Journal of Polymer Science Part B: Polymer Physics* **2008**, *46* (20), 2173–2187. https://doi.org/10.1002/polb.21550.
17. Gregorio, R., Jr.; Cestari, M. Effect of Crystallization Temperature on the Crystalline Phase Content and Morphology of Poly(Vinylidene Fluoride). *Journal of Polymer Science Part B: Polymer Physics* **1994**, *32* (5), 859–870. https://doi.org/10.1002/polb.1994.090320509.
18. Peng, G.; Zhao, X.; Zhan, Z.; Ci, S.; Wang, Q.; Liang, Y.; Zhao, M. New Crystal Structure and Discharge Efficiency of Poly(Vinylidene Fluoride-Hexafluoropropylene)/Poly(Methyl Methacrylate) Blend Films. *RSC Adv.*
19. Li, Y.; Hu, J.; He, J.; Gao, L. The Graphene Oxide Polymer Composites with High Breakdown Field Strength and Energy Storage Ability. In *The Graphene Oxide Polymer Composites with High Breakdown Field Strength and Energy Storage Ability | SpringerLink*; Springer, Cham: Turkey, 2013; Vol. 1, pp 431–438.
20. Feng, Y.; Li, W. L.; Hou, Y. F.; Yu, Y.; Cao, W. P.; Zhang, T. D.; Fei, W. D. Enhanced Dielectric Properties of PVDF-HFP/BaTiO3-Nanowire Composites Induced by Interfacial Polarization and Wire-Shape. *J. Mater. Chem. C* **2015**, *3* (6), 1250–1260. https://doi.org/10.1039/C4TC02183E.
21. Salimi, A.; Yousefi, A. A. Analysis Method: FTIR Studies of β-Phase Crystal Formation in Stretched PVDF Films. *Polymer Testing* **2003**, *22* (6), 699–704. https://doi.org/10.1016/S0142-9418(03)00003-5.
22. Li, J.; Wang, H.; Li, S. Thermal Stability and Flame Retardancy of an Epoxy Resin Modified with Phosphoric Triamide and Glycidyl POSS. *High Performance Polymers* **2019**, *31* (9–10), 1217–1225. https://doi.org/10.1177/0954008319843979.
23. Dias, J. C.; Correia, D. M.; Costa, C. M.; Ribeiro, C.; Maceiras, A.; Vilas, J. L.; Botelho, G.; de Zea Bermudez, V.; Lanceros-Mendez, S. Improved Response of Ionic Liquid-Based Bending Actuators by Tailored Interaction with the Polar Fluorinated Polymer Matrix. *Electrochimica Acta* **2019**, *296*, 598–607. https://doi.org/10.1016/j.electacta.2018.11.049.
24. Hu, Y.; Xu, P.; Gui, H.; Yang, S.; Ding, Y. Effect of Graphene Modified by a Long Alkyl Chain Ionic Liquid on Crystallization Kinetics Behavior of Poly(Vinylidene Fluoride). *RSC Adv.* **2015**, *5* (112), 92418–92427. https://doi.org/10.1039/C5RA17169E.
25. Guo, H.; Zhang, Y.; Xue, F.; Cai, Z.; Shang, Y.; Li, J.; Chen, Y.; Wu, Z.; Jiang, S. In-Situ Synchrotron SAXS and WAXS Investigations on Deformation and α–β Transformation of Uniaxial Stretched Poly(Vinylidene Fluoride). *CrystEngComm* **2013**, *15* (8), 1597–1606. https://doi.org/10.1039/C2CE26578H.
26. Bidsorkhi, H. C.; D'Aloia, A. G.; De Bellis, G.; Proietti, A.; Rinaldi, A.; Fortunato, M.; Ballirano, P.; Bracciale, M. P.; Santarelli, M. L.; Sarto, M. S. Nucleation Effect of Unmodified Graphene Nanoplatelets on PVDF/GNP Film Composites. *Materials Today Communications* **2017**, *11*, 163–173. https://doi.org/10.1016/j.mtcomm.2017.04.001.
27. Rowe, M. C.; Brewer, B. J. AMORPH: A Statistical Program for Characterizing Amorphous Materials by X-Ray Diffraction. *Computers & Geosciences* **2018**, *120*, 21–31. https://doi.org/10.1016/j.cageo.2018.07.004.
28. Esterly, D. M.; Love, B. J. Phase Transformation to β-Poly(Vinylidene Fluoride) by Milling. *Journal of Polymer Science Part B: Polymer Physics* **2004**, *42* (1), 91–97. https://doi.org/10.1002/polb.10613.

29. Cataldi, P.; Bayer, I. S.; Cingolani, R.; Marras, S.; Chellali, R.; Athanassiou, A. A Thermochromic Superhydrophobic Surface. *Sci Rep* **2016**, *6* (1), 27984. https://doi.org/10.1038/srep27984.
30. Liu, X.; Liang, B.; Long, J. Preparation of Novel Thick Sheet Graphene and Its Effect on the Properties of Polyolefins with Different Crystallinities. *Polym. Bull.* **2021**. https://doi.org/10.1007/s00289-021-03791-x.
31. Tripathi, M.; Bobade, S. M.; Kumar, A. Preparation of Polyvinylidene Fluoride-Co-Hexafluoropropylene-Based Polymer Gel Electrolyte and Its Performance Evaluation for Application in EDLCs. *Bull Mater Sci* **2019**, *42* (1), 27. https://doi.org/10.1007/s12034-018-1685-0.
32. Parangusan, H.; Ponnamma, D.; Al-Maadeed, M. A. A. Stretchable Electrospun PVDF-HFP/Co-ZnO Nanofibers as Piezoelectric Nanogenerators. *Sci Rep* **2018**, *8* (1), 754. https://doi.org/10.1038/s41598-017-19082-3.
33. Hayes, R.; Warr, G. G.; Atkin, R. Structure and Nanostructure in Ionic Liquids. *Chem. Rev.* **2015**, *115* (13), 6357–6426. https://doi.org/10.1021/cr500411q.
34. Thomas, E.; Parvathy, C.; Balachandran, N.; Bhuvaneswari, S.; Vijayalakshmi, K. P.; George, B. K. PVDF-Ionic Liquid Modified Clay Nanocomposites: Phase Changes and Shish-Kebab Structure. *Polymer* **2017**, *115*, 70–76. https://doi.org/10.1016/j.polymer.2017.03.026.
35. Sownthari, K.; Austin Suthanthiraraj, S. Preparation and Properties of a Gel Polymer Electrolyte System Based on Poly-ε-Caprolactone Containing 1-Ethyl-3-Methylimidazolium Bis(Trifluoromethylsulfonyl)Imide. *Journal of Physics and Chemistry of Solids* **2014**, *75* (6), 746–751. https://doi.org/10.1016/j.jpcs.2014.02.003.
36. Kang, S. J.; Park, Y. J.; Hwang, J. Y.; Jeong, H. J.; Lee, J. S.; Kim, K. J.; Kim, H.-C.; Huh, J.; Park, C. Localized Pressure-Induced Ferroelectric Pattern Arrays of Semicrystalline Poly(Vinylidene Fluoride) by Microimprinting. *Advanced Materials* **2007**, *19* (4), 581–586. https://doi.org/10.1002/adma.200601474.
37. Xu, P.; Cui, Z.-P.; Ruan, G.; Ding, Y.-S. Enhanced Crystallization Kinetics of PLLA by Ethoxycarbonyl Ionic Liquid Modified Graphene. *Chin J Polym Sci* **2019**, *37* (3), 243–252. https://doi.org/10.1007/s10118-019-2192-5.
38. Maiz, J.; Martin, J.; Mijangos, C. Confinement Effects on the Crystallization of Poly(Ethylene Oxide) Nanotubes. *Langmuir* **2012**, *28* (33), 12296–12303. https://doi.org/10.1021/la302675k.
39. Shalu; Kumar Singh, V.; Kumar Singh, R. Development of Ion Conducting Polymer Gel Electrolyte Membranes Based on Polymer PVdF-HFP, BMIMTFSI Ionic Liquid and the Li-Salt with Improved Electrical, Thermal and Structural Properties. *Journal of Materials Chemistry C* **2015**, *3* (28), 7305–7318. https://doi.org/10.1039/C5TC00940E.
40. Gonçalves, R.; Martins, P. M.; Caparrós, C.; Martins, P.; Benelmekki, M.; Botelho, G.; Lanceros-Mendez, S.; Lasheras, A.; Gutiérrez, J.; Barandiarán, J. M. Nucleation of the Electroactive β-Phase, Dielectric and Magnetic Response of Poly(Vinylidene Fluoride) Composites with Fe2O3 Nanoparticles. *Journal of Non-Crystalline Solids* **2013**, *361*, 93–99. https://doi.org/10.1016/j.jnoncrysol.2012.11.003.
41. Tong, Y.; Que, M.; Su, S.; Chen, L. Design of Amphiphilic Poly(Vinylidene Fluoride-Co-Hexafluoropropylene)-Based Gel Electrolytes for High-Performance Lithium-Ion Batteries. *Ionics* **2016**, *22* (8), 1311–1318. https://doi.org/10.1007/s11581-016-1662-9.
42. Wu, L.; Huang, G.; Hu, N.; Fu, S.; Qiu, J.; Wang, Z.; Ying, J.; Chen, Z.; Li, W.; Tang, S. Improvement of the Piezoelectric Properties of PVDF-HFP Using AgNWs. *RSC Adv.* **2014**, *4* (68), 35896–35903. https://doi.org/10.1039/C4RA03382E.
43. Chacko, S. K.; Rahul, M. T.; Raneesh, B.; Kalarikkal, N. Enhanced Magnetoelectric Coupling and Dielectric Constant in Flexible Ternary Composite Electrospun Fibers of PVDF-HFP Loaded with Nanoclay and NiFe2O4 Nanoparticles. *New J. Chem.* **2020**, *44* (26), 11356–11364. https://doi.org/10.1039/D0NJ02494E.
44. Kim, K. M.; Park, N.-G.; Ryu, K. S.; Chang, S. H. Characteristics of PVdF-HFP/TiO2 Composite Membrane Electrolytes Prepared by Phase Inversion and Conventional Casting Methods. *Electrochimica Acta* **2006**, *51* (26), 5636–5644. https://doi.org/10.1016/j.electacta.2006.02.038.
